# Divergent Activity Profiles of Type 1 Ryanodine Receptor Channels Carrying Malignant Hyperthermia and Central Core Disease Mutations in the Amino-Terminal Region

**DOI:** 10.1371/journal.pone.0130606

**Published:** 2015-06-26

**Authors:** Takashi Murayama, Nagomi Kurebayashi, Toshiko Yamazawa, Hideto Oyamada, Junji Suzuki, Kazunori Kanemaru, Katsuji Oguchi, Masamitsu Iino, Takashi Sakurai

**Affiliations:** 1 Department of Cellular and Molecular Pharmacology, Juntendo University Graduate School of Medicine, Tokyo 113–8421, Japan; 2 Department of Molecular Physiology, Jikei University School of Medicine, Tokyo 105–8461, Japan; 3 Department of Pharmacology, School of Medicine, Showa University, Tokyo 142–8555, Japan; 4 Department of Pharmacology, Graduate School of Medicine, The University of Tokyo, Tokyo 113–0033, Japan; Cinvestav-IPN, MEXICO

## Abstract

The type 1 ryanodine receptor (RyR1) is a Ca^2+^ release channel in the sarcoplasmic reticulum of skeletal muscle and is mutated in several diseases, including malignant hyperthermia (MH) and central core disease (CCD). Most MH and CCD mutations cause accelerated Ca^2+^ release, resulting in abnormal Ca^2+^ homeostasis in skeletal muscle. However, how specific mutations affect the channel to produce different phenotypes is not well understood. In this study, we have investigated 11 mutations at 7 different positions in the amino (N)-terminal region of RyR1 (9 MH and 2 MH/CCD mutations) using a heterologous expression system in HEK293 cells. In live-cell Ca^2+^ imaging at room temperature (~25 °C), cells expressing mutant channels exhibited alterations in Ca^2+^ homeostasis, i.e., an enhanced sensitivity to caffeine, a depletion of Ca^2+^ in the ER and an increase in resting cytoplasmic Ca^2+^. RyR1 channel activity was quantitatively evaluated by [^3^H]ryanodine binding and three parameters (sensitivity to activating Ca^2+^, sensitivity to inactivating Ca^2+^ and attainable maximum activity, i.e., gain) were obtained by fitting analysis. The mutations increased the gain and the sensitivity to activating Ca^2+^ in a site-specific manner. The gain was consistently higher in both MH and MH/CCD mutations. Sensitivity to activating Ca^2+^ was markedly enhanced in MH/CCD mutations. The channel activity estimated from the three parameters provides a reasonable explanation to the pathological phenotype assessed by Ca^2+^ homeostasis. These properties were also observed at higher temperatures (~37 °C). Our data suggest that divergent activity profiles may cause varied disease phenotypes by specific mutations. This approach should be useful for diagnosis and treatment of diseases with mutations in RyR1.

## Introduction

The type 1 ryanodine receptor (RyR1) is a Ca^2+^ release channel in the sarcoplasmic reticulum (SR) of skeletal muscle and plays an important role in excitation–contraction (E-C) coupling [1, 2]. RyR1 is a homotetramer of large (>5,000 residues) subunits. Most parts of the amino (N)-terminal cytoplasmic domain constitute the "foot" structure, which spans the junctional gap between the SR and transverse (T)-tubule, whereas a small carboxyl (C)-terminal domain (≈500 residues) contains the transmembrane segments that form an ion-conducting pore [3]. RyR1 is activated by a conformational change of the voltage sensor in the dihydropyridine receptor (DHPR) upon depolarization of the T-tubule membrane, which is referred to as the depolarization-induced Ca^2+^ release (DICR) [4, 5]. The channel is also activated by Ca^2+^, i.e., Ca^2+^-induced Ca^2+^ release (CICR) [6, 7], although the rate of Ca^2+^ release via CICR is reported to be much lower than that via DICR under physiological conditions [7, 8].

Mutations in RyR1 are associated with several muscle disorders, including malignant hyperthermia (MH) and central core disease (CCD) [9]. MH is an autosomal dominant and potentially lethal pharmacogenetic disorder in which the inhalation of volatile anesthetics (e.g., halothane) or muscle relaxants (e.g., succinylcholine) triggers high fever and muscle contracture, leading to death if untreated [10]. More than 150 different point mutations for MH have been identified in the RyR1 gene and the majority of mutations cluster in three 'hotspots': N-terminal (35–614) and central (2129–2458) regions located in the cytoplasm, and carboxyl (C)-terminal region (4637–4973) near or within channel forming segments [11]. CCD is an autosomal dominant myopathy that is characterized by hypotonia at birth, mild delay in childhood development and skeletal malformations [12]. More than 60 mutations for CCD have been identified in the RyR1 gene and these mutations are also clustered in similar hotspots to the MH mutations [11]. CCD mutations in the N-terminal and central regions are susceptible to MH (i.e., MH/CCD mutations) [9]. The underlying pathology of these mutations is an enhanced Ca^2+^ release activity of the RyR1 channel, i.e., gain-of-function phenotype. In MH patients, accelerated channel activation leads to increased sensitivity to drugs (halothane or succinylcholine). In MH/CCD patients, the accelerated channel activity triggers uncompensated Ca^2+^ leakage from the SR under resting conditions [13–15]. Some CCD mutations in the C-terminal region, in contrast, cause loss-of-function phenotype, in which Ca^2+^ release triggered by depolarization is strongly suppressed (E-C uncoupling) [16–18]. These CCD mutations appear to be insusceptible to MH.

RyR1 exhibits biphasic bell-shaped Ca^2+^ dependence in CICR [6, 7]. This is explained by the action of two distinct Ca^2+^ sites: binding of Ca^2+^ to a high-affinity site (A-site) activates the channel, whereas binding of Ca^2+^ to a low-affinity site (I-site) inactivates the channel. Ca^2+^ sensitivities of A- and I-sites are important parameters for the activity of the RyR1 channel. In addition, a third parameter, i.e., gain, sets the maximal attainable activity independent of Ca^2+^ sensitivities [8, 19]. The three parameters define the activity profiles of the RyR1 channel. However, it remains unclear how disease-associated mutations affect the activity profiles and which parameter(s) of the activity profiles is important for different phenotypes, i.e., MH and MH/CCD.

In this study, we investigated 11 mutations at 7 different positions in the amino (N)-terminal region of RyR1 (9 MH and 2 MH/CCD mutations) using a heterologous expression system in HEK293 cells. The disease-associated mutations divergently altered the activity profiles in a site-specific manner by increasing the gain and the sensitivity to activating Ca^2+^. Our results provide a reasonable explanation for the mechanisms of MH and MH/CCD phenotypes caused by specific mutations in the N-terminal region of the RyR1 channel.

## Materials and Methods

### Construction of the expression plasmids

cDNA cassettes encoding the full-length rabbit skeletal muscle RyR1 (pBS-RyR1) were used for mutagenesis [20]. Each disease-associated mutation in the N-terminal region corresponding to C36R, R164C, R164L, G249R, G342R, R402C, R402H, Y523C, Y523S, R615C and R615L was introduced by inverse polymerase chain reaction (PCR) using a *Hind*III-*Sal*I fragment (pBS-RyR1cs1) or a *Sal*I-*Bsu*36I fragment (pBS-RyR1cs2) as the PCR template. The mutations were confirmed by DNA sequencing. Each mutated fragment was subcloned into the expression vector (pcDNA5/FRT/TO-RyR1) [21] using the appropriate restriction enzymes.

### Generation of stable inducible HEK293 cell lines

HEK293 cells stably and inducibly expressing RyR1 mutants were generated as described [21] using the Flp-In T-REx system (Life Technologies, CA, USA). Clones with suitable expression of RyR1 were selected and used for experiments.

### Single-cell Ca^2+^ imaging

Ca^2+^ measurements were carried out in HEK293 cells expressing WT or mutant RyR1 grown on a glass bottom dish for 24–30 h after induction by doxycycline. Experiments were carried out at room temperature (RT; ~25°C) or 36°C using a temperature control system for glass bottom dishes (CL-100/SC29 and TC324C, Warner Instruments, CT, USA).

#### Determination of caffeine responses with Fluo-4

For determination of caffeine responses, HEK293 cells were loaded with 4 μM fluo-4 AM in culture medium for 30 min at 37°C in a CO_2_ incubator and then washed with HEPES buffered Krebs solution (140 mM NaCl, 5 mM KCl, 2 mM CaCl_2_, 1 mM MgCl_2_, 11 mM glucose, 5 mM HEPES, pH 7.4). The dish was placed on the stage of an inverted microscope equipped with the Nipkow disc confocal system (CSU22, Yokogawa, Japan). Cells were treated initially with 10 mM caffeine to deplete Ca^2+^ and then equilibrated with normal Krebs solution for 5 min. The cells were then perfused with caffeine-containing (0.1–10 mM) Krebs solution, as described previously [21] and fluo-4 signals, excited at 488 nm and emitting at 525 nm, were captured with a electron-multiplying (EM)-CCD camera at 700 ms intervals (Model 8509, Hamamatsu Photonics, Hamamatsu, Japan). At the end of each experiment, a high Ca^2+^ Krebs solution containing 20 mM Ca^2+^ and 20 μM ionomycin was applied to obtain the maximal fluorescence intensity (*F*_max_) of fluo-4 in individual cells. Average fluorescence intensities in individual cells were determined using region of interest (ROI) analysis with AquaCosmos software and normalized to the *F*_max_.

#### Resting cytoplasmic Ca^2+^ measurements with Fura-2

For measurement of resting cytoplasmic Ca^2+^ ([Ca^2+^]_i_), cells were loaded at RT with 4 μM fura-2 AM for 30 min in a physiological salt solution (PSS) containing 150 mM NaCl, 4 mM KCl, 2 mM CaCl_2_, 1 mM MgCl_2_, 5.6 mM glucose and 10 mM HEPES at pH 7.4. Cells were treated initially with 10 mM caffeine to deplete Ca^2+^ and then equilibrated in PSS for 10 min. Fluorescence images were acquired at >420 nm using an inverted microscope equipped with a cooled CCD camera at a rate of one frame every 2 s. Excitation wavelengths were 345 nm and 380 nm. Ca^2+^ imaging experiments were conducted at RT. Image analysis was carried out using IPLab software (BD Biosciences Bioimaging, MD, USA). ROIs corresponding to individual cells were selected and the average fluorescence intensity (*F*) of each ROI minus the background intensity was calculated for each frame. We used the *F*_345_/*F*_380_ ratio (the value of *F* at an excitation wavelength of 345 nm divided by the value of *F* at an excitation wavelength of 380 nm) to estimate [Ca^2+^]_i_, as described previously [22]. The *K*_D_ (239 nM) for Ca^2+^ was determined via an *in vitro* calibration of fura-2 fluorescence.

#### Simultaneous recordings of ER and cytoplasmic Ca^2+^

For simultaneous recording of ER and cytoplasmic Ca^2+^, cells were co-transfected with genetically-encoded Ca^2+^ indicators, G-GECO1.1 [23] for [Ca^2+^]_i_ and R-CEPIA1er [24] for ER Ca^2+^ ([Ca^2+^]_ER_). Doxycycline was added to the culture medium at the time of transfection. At 24–30 h after transfection, cells were washed with the HEPES-buffered Krebs solution and placed on the stage of the microscope. G-GECO1.1 and R-CEPIA1er were excited by 488 nm and 568 nm laser light, respectively, and fluorescence images at 525 and 620 nm were simultaneously captured, side-by-side on the same camera, using the W-view system (Hamamatsu Photonics, Hamamatsu, Japan). At the end of each experiment, the high Ca^2+^ Krebs solution was applied to obtain the *F*_max_ of the Ca^2+^ indicators in individual cells. The fraction of the Ca^2+^-independent basal fluorescence signal was determined separately in the presence of 5 mM 1,2-bis(o-aminophenoxy)ethane-N,N,N',N'-tetraacetic acid (BAPTA) plus 20 μM ionomycin and subtracted from the total fluorescence signal. [Ca^2+^]_ER_ was calculated using parameters obtained by an *in situ* titration (*K*_D_ = 455 nM, *n* = 1.59) [24].

### [^3^H]Ryanodine binding assay

HEK293 cells were cultured in five 150 mm dishes, and protein expression was induced with doxycycline (2 μg/ml) for 48 h. Microsomes were prepared by nitrogen cavitation [21]. The microsomes (50–100 μg of protein) were incubated with 5 nM [^3^H]ryanodine for 5 h at 25°C in a 100 μl solution containing 0.17 M NaCl, 20 mM 3-(*N*-morpholino)-2-hydroxypropanesulfonic acid (MOPSO), pH 6.8, 2 mM dithiothreitol, 1 mM AMP and various concentrations of free Ca^2+^ buffered with 10 mM ethylene glycol-bis(2-aminoethylether)-N,N,N',N'-tetraacetic acid (EGTA). For [^3^H]ryanodine binding experiments at 37°C, the incubation period was shortened to 3 h. Ca^2+^ concentrations were calculated using MaxChelator (http://maxchelator.stanford.edu) [25]. The protein-bound [^3^H]ryanodine was separated by filtering through polyethyleneimine-treated Whatman GF/B glass filters. Nonspecific binding was determined in the presence of 20 μM unlabeled ryanodine. The [^3^H]ryanodine binding data (*B*) were normalized by the maximum number of functional channels (*B*_max_), which was separately determined by Scatchard plot analysis using varied concentrations (3–20 nM) of [^3^H]ryanodine in a high-salt buffer containing 1 M NaCl [8]. The resultant *B*/*B*_max_ represents the averaged activity of each mutant.

### Parameter analysis

To obtain the parameters of Ca^2+^-dependent [^3^H]ryanodine binding, the data were fitted to the following equation:

$$A=A_{\text{max}}\times f_{\text{A}}\times (1-f_{\text{I}})$$ (1)

where *A* is the activity at the specified Ca^2+^, *A*_max_ is the gain that determines the maximal attainable activity, and *f*_A_ and *f*_I_ are fractions of the activating Ca^2+^ site (A-site) and inactivating Ca^2+^ site (I-site) occupied by Ca^2+^, respectively [8]. *f*_A_ and *f*_I_ at the specified Ca^2+^ concentration ([Ca^2+^]) are expressed as:

$$f_{\text{A}}={\left[{\text{Ca}}^{2+}\right]}^{n_{\text{A}}}/({\left[{\text{Ca}}^{2+}\right]}^{n_{\text{A}}}+K_{\text{A}}{}^{n_{\text{A}}})$$ (2)

$$f_{\text{I}}={\left[{\text{Ca}}^{2+}\right]}^{n_{\text{I}}}/({\left[{\text{Ca}}^{2+}\right]}^{n_{\text{I}}}+K_{\text{I}}{}^{n_{\text{I}}})$$ (3)

where *K*_A_ and *K*_I_ are dissociation constants, and *n*_A_ and *n*_I_ are Hill coefficients for Ca^2+^ of A- and I-sites, respectively. From the shape of the Ca^2+^-dependent curves for WT and mutant channels, we assumed that the Hill coefficients are not largely dissimilar between the WT and mutant channels. We therefore used fixed values for *n*_A_ (1.2) and *n*_I_ (1.5) for WT and all the mutants at both 25 and 37°C. These values were chosen so as to maximize the sum of *R* ^2^ values for curve fitting of WT and all mutant channels. Curve fitting was performed using the Prism 6 software (GraphPad Software, CA, USA). The curves for *f*_A_, 1–*f*_I_, and *A* are shown in S1 Fig.

For estimation of ryanodine binding at resting [Ca^2+^]_i_, *A* at pCa 7 for each mutant was calculated by eqs (1)–(3) using the obtained parameters (*K*_A_, *K*_I_ and *A*_max_).

### Western blotting

Microsomal proteins were separated by sodium dodecyl sulfate polyacrylamide gel electrophoresis and transferred onto a polyvinylidene fluoride membrane. Western blotting was performed using antibodies for RyR1 (34C, Developmental Studies Hybridoma Bank, University of Iowa, IA, USA) and calnexin (C4731, Sigma-Aldrich, MO, USA).

### Data analysis

Data are presented as the means ± SE. Statistical comparisons have been made using the Prism 6 software. Student’s t-test was used to compare two groups. *P* values < 0.05 were considered significant.

## Results

### Expression of RyR1 mutants

We chose 11 mutations at 7 different positions in the N-terminal region, one of the hotspots of disease-associated mutations [11] (Table 1). Two different mutations were made at four positions (R164C/R164L, R402C/R402H, Y523C/Y523S and R615C/R615L). All mutations are susceptible to MH and two of them (R164C and Y523S) are also reported to show the CCD phenotype (i.e., MH/CCD) [9]. Six positions (C36, R164, G249, G342, R402 and Y523) map to the N-terminal region in the crystal structure and are localized to different domains/interfaces of the structure [26]. Functional characterizations using heterologous expression systems have been done for most mutations except for R402H and Y523C (see References in Table 1). Among the 11 mutations, animal models exist for three mutations: R163C knock-in mice [27], Y524S knock-in mice [28] and MH pigs carrying R615C [29].

**Table 1:** Disease-associated mutations used in this study.

Residue^a^	Disease	Domain^b^	Interface^b^	Functional characterizationin expression system (Refs)
C36R	MH	A	A-B	[20, 31]
R164C	MH/CCD	A	Interface 1, 4	[14, 20, 31, 44–46, 59]
R164L	MH	A	Interface 1, 4	[42]
G249R	MH	B	Buried	[20, 31, 59]
G342R	MH	B	Interface 5	[20, 31, 44]
R402C	MH	C	A-C	[54]
R402H	MH	C	A-C	–
Y523C	MH	C	interface 3	–
Y523S	MH/CCD	C	interface 3	[14, 20, 30, 31]
R615C	MH	–	–	[15, 20, 30, 31, 44, 45]
R615L	MH	–	–	[20, 31]

^a^Residue numbers refer to rabbit RyR1.

^b^Domain and interface information is from the crystal structure of the RyR1 ABC domains [26].

Wild-type (WT) and mutant RyR1s were heterologously expressed in HEK293 cells using a tetracycline-inducible expression system [21]. The RyR1 mutants were expressed at similar levels to WT (S2 Fig).

### Caffeine-induced Ca^2+^ transients

**Fig 1:**
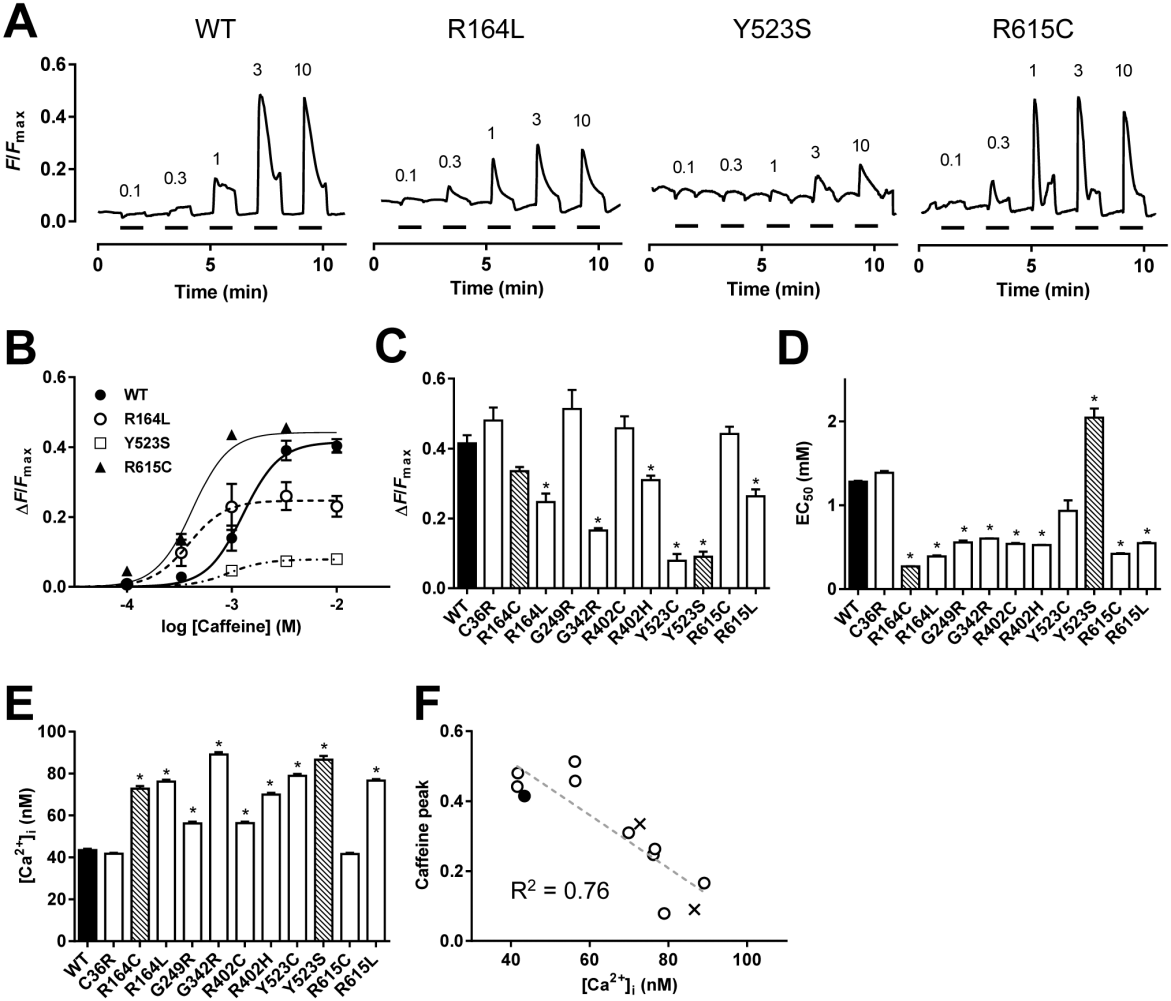
Caffeine-induced Ca^2+^ transients in cells expressing WT and mutant RyR1s. **A–D**. HEK293 cells expressing WT or mutant RyR1 channels were loaded with fluo-4 AM and stimulated by different concentrations (0.1–10 mM) of caffeine. Measurements were carried out at room temperature (RT). **A.** Representative traces of fluo-4 signals for WT and three mutants (R164L, Y523S and R615C). Caffeine was applied at the time points indicated by the short horizontal bars. Fluo-4 signals were normalized by *F*_max_ (see Materials and Methods). **B.** The magnitude of the Ca^2+^ transients were plotted against caffeine concentrations and fitted to the dose-response curve. **C** and **D.** The maximum Ca^2+^ transients **(C)** and EC_50_ for caffeine **(D)** of WT (filled column), MH mutations (open columns) and MH/CCD mutations (hatched columns). Data are means ± SE (*n* = 78–150). **E.** HEK293 cells of WT (filled column), MH mutations (open columns) and MH/CCD mutations (hatched columns) were loaded with fura-2 AM and resting [Ca^2+^]_i_ was determined. Data are means ± SE (*n* = 207–494). **F.** The maximum caffeine-induced Ca^2+^ transients correlate well with resting [Ca^2+^]_i_. (*R* ^2^ = 0.76, dashed line).

To test the phenotypes of RyR1 with disease-associated mutations, we initially examined caffeine-induced Ca^2+^ transients in HEK293 cells at RT (25°C) (Fig 1). This assay is based on the observation that caffeine increases the sensitivity of RyR1 to activating Ca^2+^ [1, 2] and the EC_50_ for caffeine is a marker for detecting the MH phenotype. Typical fluo-4 Ca^2+^ signals are shown in Fig 1A. In WT, Ca^2+^ transients were detected at ~0.3 mM caffeine and reached a plateau at 3 mM or higher concentrations. Disease-associated mutants showed divergent responses. R164L produced Ca^2+^ transients at lower caffeine concentrations than WT with a reduced peak height. The MH/CCD phenotype Y523S exhibited very small Ca^2+^ transients. R615C showed enhanced caffeine sensitivity with a peak height comparable to WT. Dose-dependent plots of caffeine-induced Ca^2+^ transients revealed increased caffeine sensitivity and/or reduced peak amplitude in disease-associated mutations (Fig 1B). We determined the maximum Ca^2+^ transients (Fig 1C) and the EC_50_ for caffeine (Fig 1D) from the dose-dependent curves of each mutation. The peak Ca^2+^ transients were smaller and varied to a degree for some mutants (R164C, R164L, G342R, R402H, Y523C, Y523S and R615L) when compared with WT, but showed no change in other mutants (C36R, G249R, R402C and R615C). All the mutants except for C36R, Y523C and Y523S exhibited a significant reduction in the EC_50_ value.

We also noticed that the resting cytoplasmic Ca^2+^ ([Ca^2+^]_i_) level was higher in some disease-associated mutations than in WT (e.g., R164L and Y523S in Fig 1A). We quantitatively measured resting [Ca^2+^]_i_ using fura-2. Resting [Ca^2+^]_i_ was around 40 nM in cells expressing WT (Fig 1E). The disease-associated mutants, except for C36R and R615C, exhibited significantly higher resting [Ca^2+^]_i_, with values ranging between 50–90 nM. There was an inverse correlation between resting [Ca^2+^]_i_ and the maximum peak Ca^2+^ transients induced by caffeine (*R* ^2^ = 0.76) (Fig 1F).

### Measurements of ER luminal Ca^2+^

**Fig 2:**
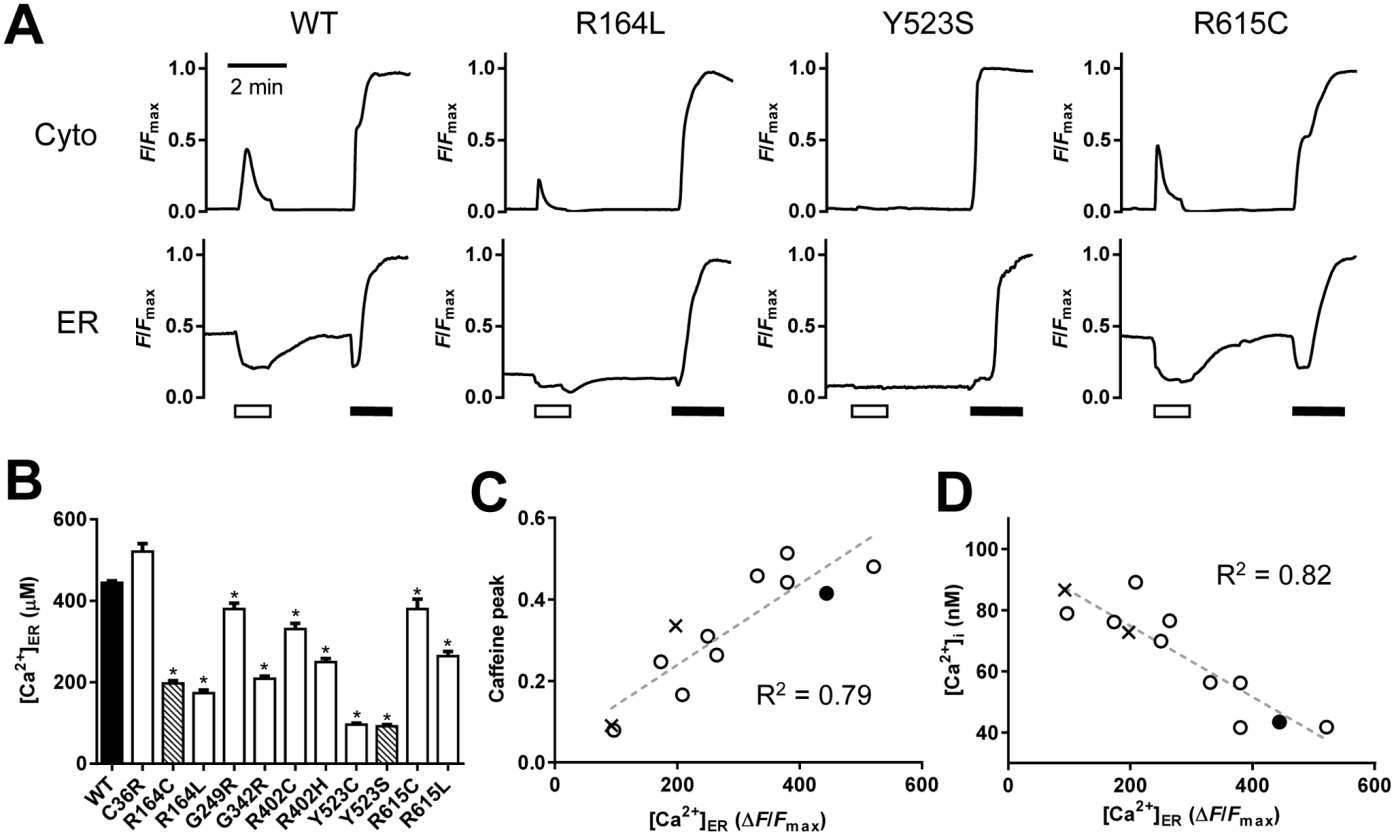
Determination of resting [Ca^2+^]_ER_ in cells expressing WT or mutant RyR1s. HEK293 cells expressing WT or mutant RyR1 channels were transfected with G-GECO1.1 and R-CEPIA1er to determine [Ca^2+^]_i_ and [Ca^2+^]_ER_, respectively. Measurements were carried out at RT. **A.** Typical traces for [Ca^2+^]_i_ (upper) and [Ca^2+^]_ER_ (lower) signals. Caffeine was initially applied to deplete Ca^2+^ in the ER (white bars) and then Ca^2+^ in the ER was determined at the plateau after the removal of caffeine. Finally, *F*_max_ for indicators was determined by the application of 20 μM ionomycin and 20 mM Ca^2+^ (black bars). **B.** [Ca^2+^]_ER_ of WT (filled column), MH mutations (open columns) and MH/CCD mutations (hatched columns). Data are means ± SE (*n* = 35–99). *p < 0.05 compared with WT. **C** and **D.** Peak caffeine-induced Ca^2+^ transients **(C)** and resting [Ca^2+^]_i_ **(D)** of WT (filled circle), MH mutations (open circles) and MH/CCD mutations (crosses) were plotted against [Ca^2+^]_ER_. Note that the peak caffeine-induced Ca^2+^ transients (*R* ^2^ = 0.79) and resting [Ca^2+^]_i_ (*R* ^2^ = 0.82) correlated strongly with [Ca^2+^]_ER_ (dashed lines).

The reduced peak value of caffeine-induced Ca^2+^ transients and corresponding increased resting [Ca^2+^]_i_ suggest a reduction of Ca^2+^ in the ER Ca^2+^ store ([Ca^2+^]_ER_). To test this possibility, we simultaneously measured [Ca^2+^]_i_ and [Ca^2+^]_ER_ in HEK293 cells (Fig 2). Fig 2A shows typical traces of [Ca^2+^]_i_ and [Ca^2+^]_ER_ signals. We initially treated the cells with 3 mM caffeine to deplete Ca^2+^ in the ER (open bars). After removal of the caffeine, [Ca^2+^]_ER_ gradually recovered and achieved a steady-state level. Resting [Ca^2+^]_ER_ was measured as *F*/*F*_max_, in which fluorescence intensity of the steady-state level (*F*) was normalized by the maximum fluorescence intensity (*F*_max_) of the indicator, which was determined by treating cells with high Ca^2+^ plus ionomycin at the end of experiments (filled bars). R164L and Y523S showed markedly lower [Ca^2+^]_ER_ than WT, whereas R615C exhibited a [Ca^2+^]_ER_ level similar to that of WT (Fig 2A). The *F*/*F*_max_ values were converted to Ca^2+^ concentrations using parameters obtained in the *in situ* titration [24] and are summarized in Fig 2B. Two MH/CCD mutations, R164C and Y523S, exhibited severe depletion of [Ca^2+^]_ER_, which is consistent with previous reports [14, 15, 30, 31]. The other mutations, except for C36R, also showed significant reduction in [Ca^2+^]_ER_. The [Ca^2+^]_ER_ strongly correlated with the peak value of caffeine-induced Ca^2+^ transients (*R* ^2^ = 0.79) (Fig 2C) and resting [Ca^2+^]_i_ (*R* ^2^ = 0.82) (Fig 2D), indicating that small peak values of caffeine-induced Ca^2+^ transients are due to low [Ca^2+^]_ER_ levels.

**Fig 3:**
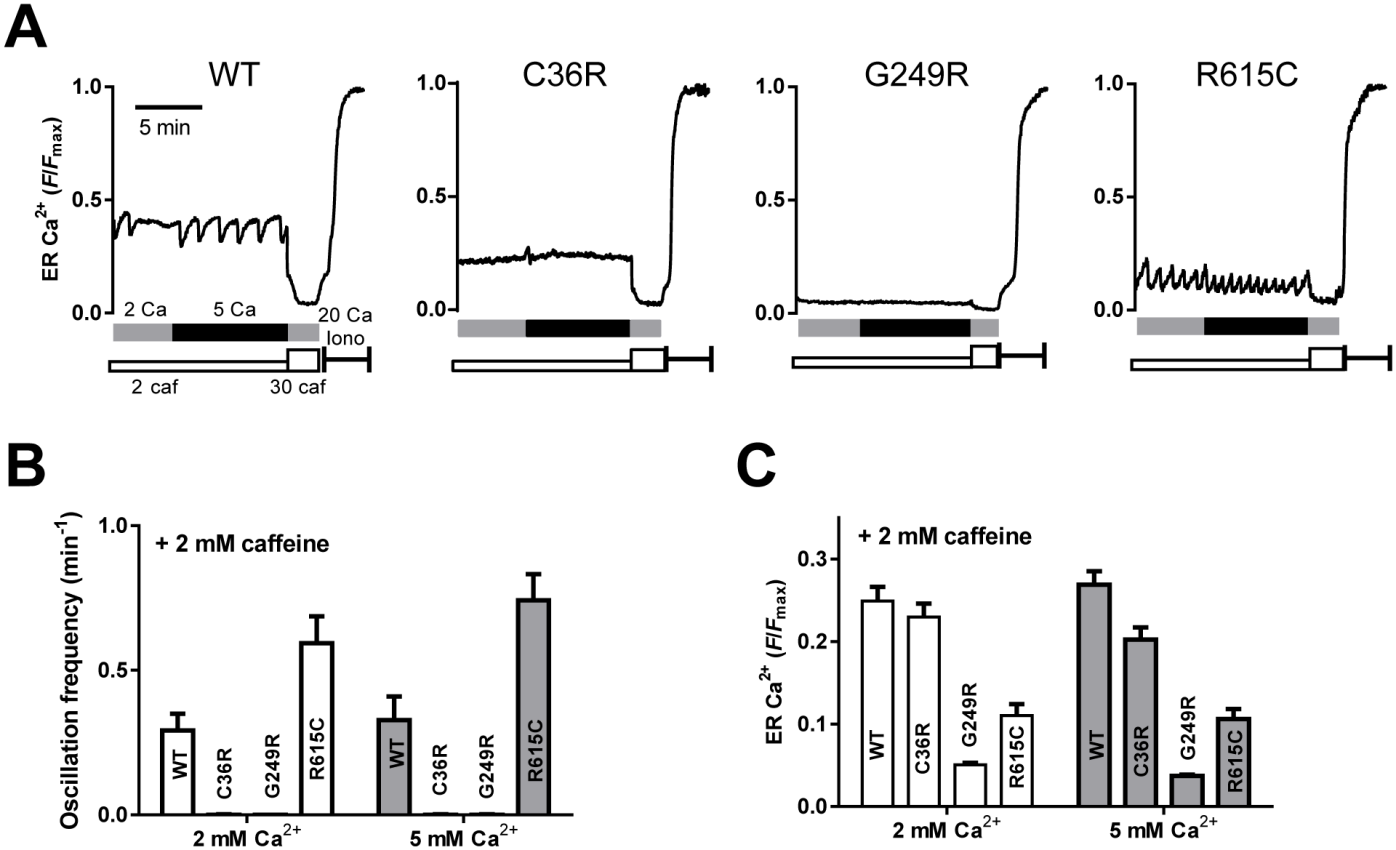
Caffeine-induced Ca^2+^ oscillations by monitoring ER Ca^2+^. ER Ca^2+^ of HEK293 cells expressing WT or mutant RyR1 channels (C36R, G249R and R615C) was monitored with R-CEPIA1er as described in Fig 2. **A.** Typical traces for ER Ca^2+^ signals. Cells were incubated for 2 min with normal Krebs solution containing 2 mM Ca^2+^ and 2 mM caffeine (grey bar). Ca^2+^ in Krebs solution was then increased to 5 mM (black bars). After depletion of Ca^2+^ by 30 mM caffeine, *F*_max_ for indicators was determined by the application of 20 μM ionomycin and 20 mM Ca^2+^. **B.** Ca^2+^ oscillation frequencies determined with normal (2 mM Ca^2+^) and 5 mM Ca^2+^ Krebs in the presence of 2 mM caffeine. **C.** Caffeine-induced Ca2+ oscillations by monitoring ER Ca2+. ER [Ca^2+^]_ER_ levels in normal and 5 mM Ca^2+^ Krebs solution with 2 mM caffeine. For WT and R615C, upper levels during Ca^2+^ oscillations (threshold levels for Ca^2+^ release) were measured.

It has been reported that caffeine or halothane induces Ca^2+^ oscillations in HEK cells expressing WT RyR1 and that a MH-associated mutation (R615C) reduced a threshold level of luminal Ca^2+^ for the spontaneous Ca^2+^ release, which is referred to as store-overload-induced Ca^2+^ release (SOICR) level [32]. We therefore tested whether this is also the case with the other disease-associated mutations by determining [Ca^2+^]_ER_ (Fig 3). Unlike RyR2 expressing cells, no Ca^2+^ oscillations were detected in WT nor any mutant RyR1 cells during 8-min observations in the absence of caffeine. In the presence of low concentration of caffeine, Ca^2+^ oscillations were detected in WT cells (9 out of 42 cells) (Fig 3A). R615C also exhibited Ca^2+^ oscillations (16 out of 36 cells) with higher frequency than that of WT (Fig 3B). These results are in agreement with the previous report [32].

The other 10 mutants, however, rarely showed Ca^2+^ oscillations; instead, they exhibited the constant [Ca^2+^]_ER_ level during the observations (see Fig 3A, C36R and G249R). The [Ca^2+^]_ER_ levels of C36R and G249R were higher and lower than that for R615C, respectively, suggesting no correlation between Ca^2+^ oscillations and [Ca^2+^]_ER_ level in the MH mutants (Fig 3C). Increase in extracellular Ca^2+^ ([Ca^2+^]_o_) from 2 mM to 5 mM did not induce Ca^2+^ oscillations (Fig 3B) nor significantly affect the [Ca^2+^]_ER_ level (Fig 3C). To summarize, high propensity for spontaneous Ca^2+^ oscillation was not a common characteristic of all MH mutations.

### Determination of the activity profiles by [^3^H]ryanodine binding and parameter analysis

**Fig 4:**
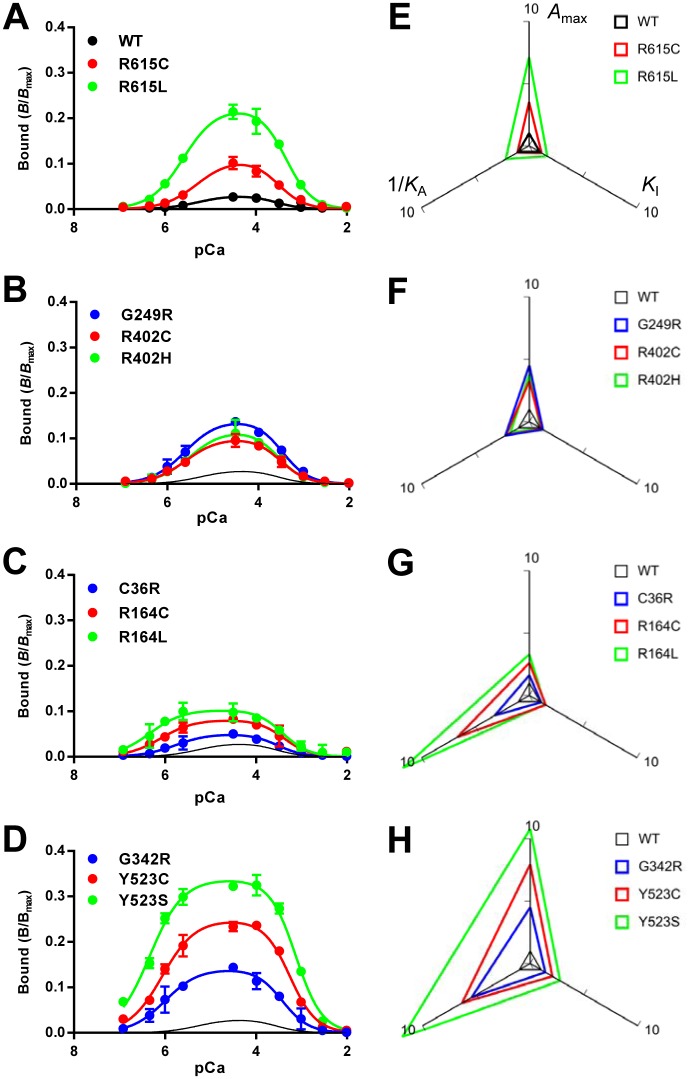
Ca^2+^-dependent [^3^H]ryanodine binding of WT and mutant RyR1s. **A–D.** Ca^2+^-dependent [^3^H]ryanodine binding was determined at 25°C in 0.17 M NaCl, 20 mM MOPSO, pH 6.8, 2 mM dithiothreitol, 1 mM AMP and various concentrations of Ca^2+^ buffered with 10 mM EGTA. Curves without data points in **B–D** indicate WT. Data are means ± SE (*n* = 3–5). Note that the mutants show greater [^3^H]ryanodine binding than WT. **E–H.** Activity profiles of the mutant channels. The three parameters, *A*_max_, *K*_A_ and *K*_I_, were obtained by fitting analysis (see Materials and Methods) and plotted on the radar charts relative to WT. 1/*K*_A_ was used as the parameter for activating Ca^2+^ dissociation constants, in which a larger value represents higher sensitivity. Note that the size of the triangle represents the magnitude of the channel activity.

To quantitatively evaluate the activity of mutant RyR1 channels, we determined Ca^2+^-dependent [^3^H]ryanodine binding using microsomes isolated from HEK293 cells. Since ryanodine specifically binds to the open channel, [^3^H]ryanodine binding is a useful measure for functional state of the RyR channel [33–35]. The [^3^H]ryanodine binding value at the specified [Ca^2+^] was expressed as *B*/*B*_max_, which represents the ratio of active to total channels under the conditions employed [8]. WT exhibited biphasic Ca^2+^-dependent [^3^H]ryanodine binding with the peak near 30 μM Ca^2+^ (Fig 4A). The peak *B*/*B*_max_ value was ≈0.03, which is consistent with RyR1 from skeletal muscle [36, 37]. All the disease-associated mutants showed greater binding than WT (Fig 4A–4D). To our surprise, there was a large variation in Ca^2+^-dependent curves between mutants.

The results of the [^3^H]ryanodine binding were fitted to eqs (1))–(3) to obtain the three parameters, i.e., the gain (*A*_max_) and dissociation constants for activating Ca^2+^ (*K*_A_) and inactivating Ca^2+^ (*K*_I_) (S1 Fig). The obtained parameters are summarized in S1 Table. *A*_max_ increased (1.6–10.8-fold) for all the mutations relative to the WT channel. The *K*_A_ greatly decreased (0.08–0.18-fold) in two MH/CCD mutations (R164C and Y523S) and three MH mutations (R164L, G342R and Y523C) when compared with the results of the WT, whereas moderate decreases (0.31–0.57-fold) in *K*_A_ were observed for the other MH mutations, except for R615C. The *K*_I_ increased (1.5–2.8-fold) in four mutants (R164C, Y523C, Y523S and R164L) when compared with the results of the WT, but no significant change in the other mutants was observed.

Activity profiles of mutant channels were visualized by radar charts, in which the three parameters were plotted relative to WT (Fig 4E–4H). 1/*K*_A_ was used as the parameter for sensitivity to activating Ca^2+^, in which a larger value represents higher sensitivity. The size of the triangle represents the magnitude of the activity. We found that two triangles for mutations at the same sites were similar in shape. In R615C and R615L, the increase in *A*_max_ was more marked when compared with 1/*K*_A_ and *K*_I_ (Fig 4E). Similar properties were also observed with R402C, R402H and G249R (Fig 4F). Mutations at R164 (R164C and R164L) and C36 (C36R), in contrast, exhibited more marked enhancement in 1/*K*_A_ compared with *A*_max_ and *K*_I_ (Fig 4G). In G342R, Y523C and Y523S, all three parameters were greatly increased (Fig 4H). These findings strongly suggest site-specific effects of the mutations on the activity profiles. The two MH/CCD mutants, R164C and Y523S, both exhibited a marked increase in 1/*K*_A_. Interestingly, some MH mutants (R164L, G342R and Y523C) showed activity profiles similar to the MH/CCD mutants (Fig 4G and 4H).

### Estimation of the activity of mutant channels at resting [Ca^2+^]_i_

**Fig 5:**
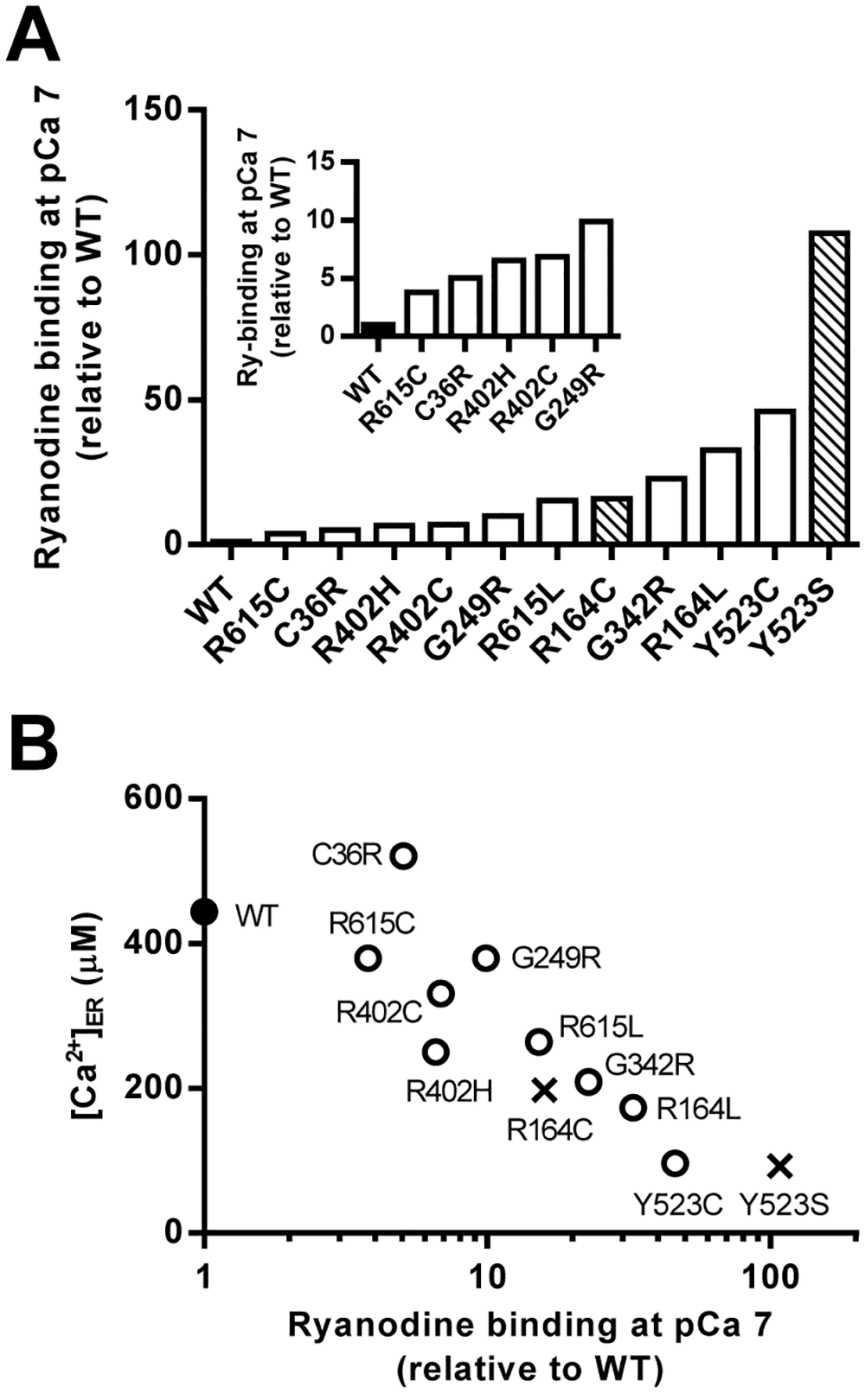
Estimation of the activity of mutant channels at resting [Ca^2+^]_i_. **A.** Ryanodine binding of mutant channels under resting conditions (pCa 7) was estimated by substituting the three parameters (*K*_A_ and *K*_I_ and *A*_max_) into eqs (1)–(3) and plotted relative to WT in ascending order. **B.** [Ca^2+^]_ER_ for WT (filled circle), MH mutations (open circles) and MH/CCD mutations (crosses) were plotted against their estimated ryanodine binding at pCa 7.

To consider the pathological phenotype by specific mutations, it is critically important to obtain quantitative information about the activity of the mutant channel at resting [Ca^2+^]_i_. However, [^3^H]ryanodine binding over the resting [Ca^2+^]_i_ range was too low to be accurately determined (see Fig 4). Instead, we estimated the ryanodine binding of the mutant channel at pCa 7 using the three parameters (*K*_A_, *K*_I_ and *A*_max_) listed in S1 Table (see Materials and Methods). Binding was significantly higher than WT with large variability (4–100-fold increase) (Fig 5A). The rank order was as follows: WT < R615C < C36R < R402H ≈ R402C < G249R < R615L ≈ R164C < G342R < R164L < Y523C < Y523S. To test the validity of these values, we compared the estimated [^3^H]ryanodine binding with the [Ca^2+^]_ER_ of HEK293 cells, which reflects Ca^2+^ leakage via Ca^2+^ release. A good inverse correlation between the two was observed (Fig 5B).

### Effects of temperature on the properties of the mutant channels

The recent crystallographic study of the N-terminal domain of RyR1 has shown that several mutations, including C36R, significantly decrease the melting point from 47°C for the WT to ~40°C (37.7°C for C36R) [38]. It is hypothesized that these mutations may destabilize the channel at the temperature of the body, but not at RT. We therefore investigated the properties of the mutant channels at higher temperatures (36–37°C).

**Fig 6:**
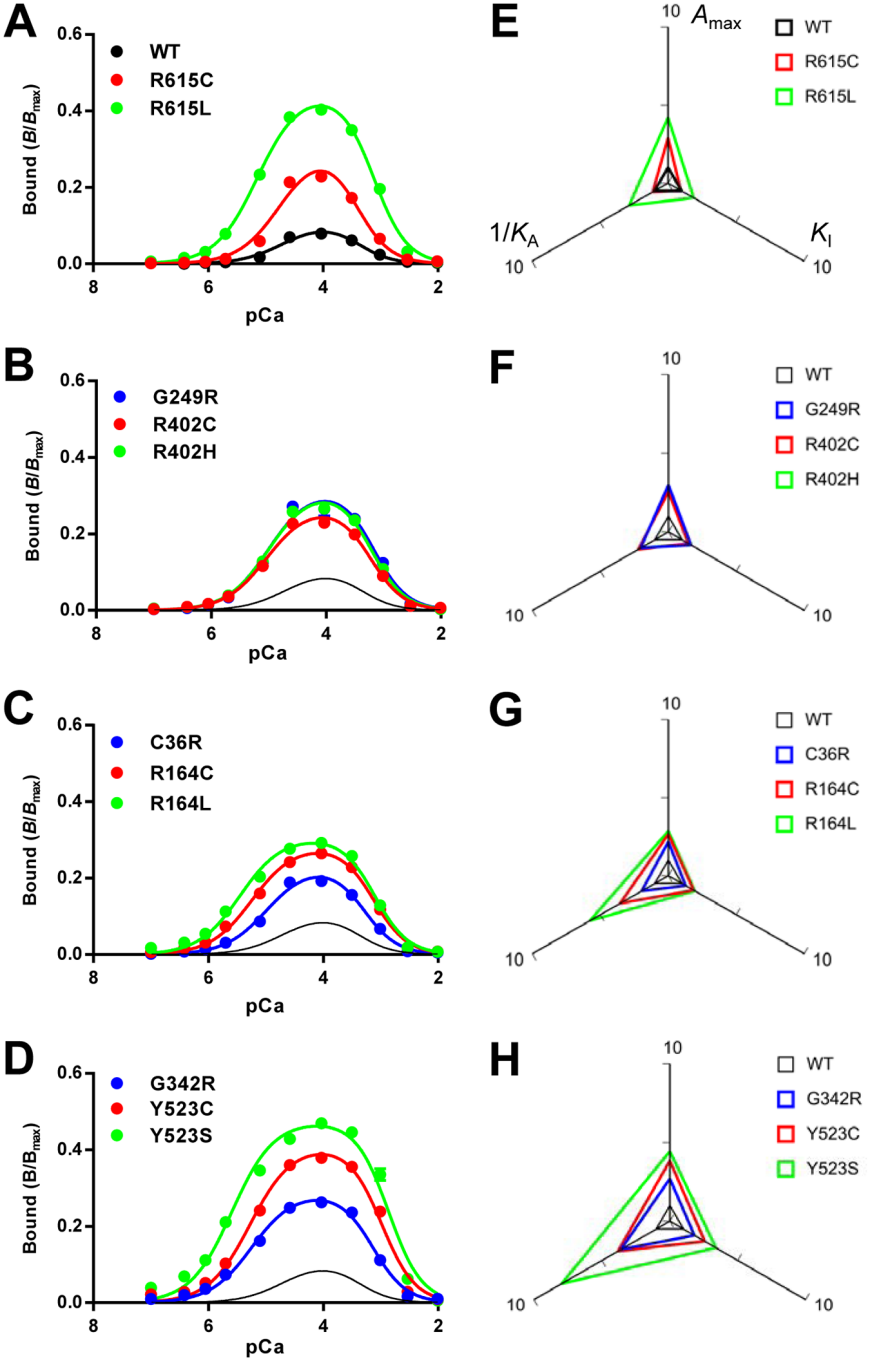
Ca^2+^-dependent [^3^H]ryanodine binding of WT and mutant RyR1s at 37°C. **A–D.** Ca^2+^-dependent [^3^H]ryanodine binding was determined as in Fig 4 at 37°C instead of RT. Curves without data points in **B–D** indicate WT. Data are means ± SE (*n* = 3–5). **E–H.** Activity profiles of the mutant channels.

**Fig 7:**
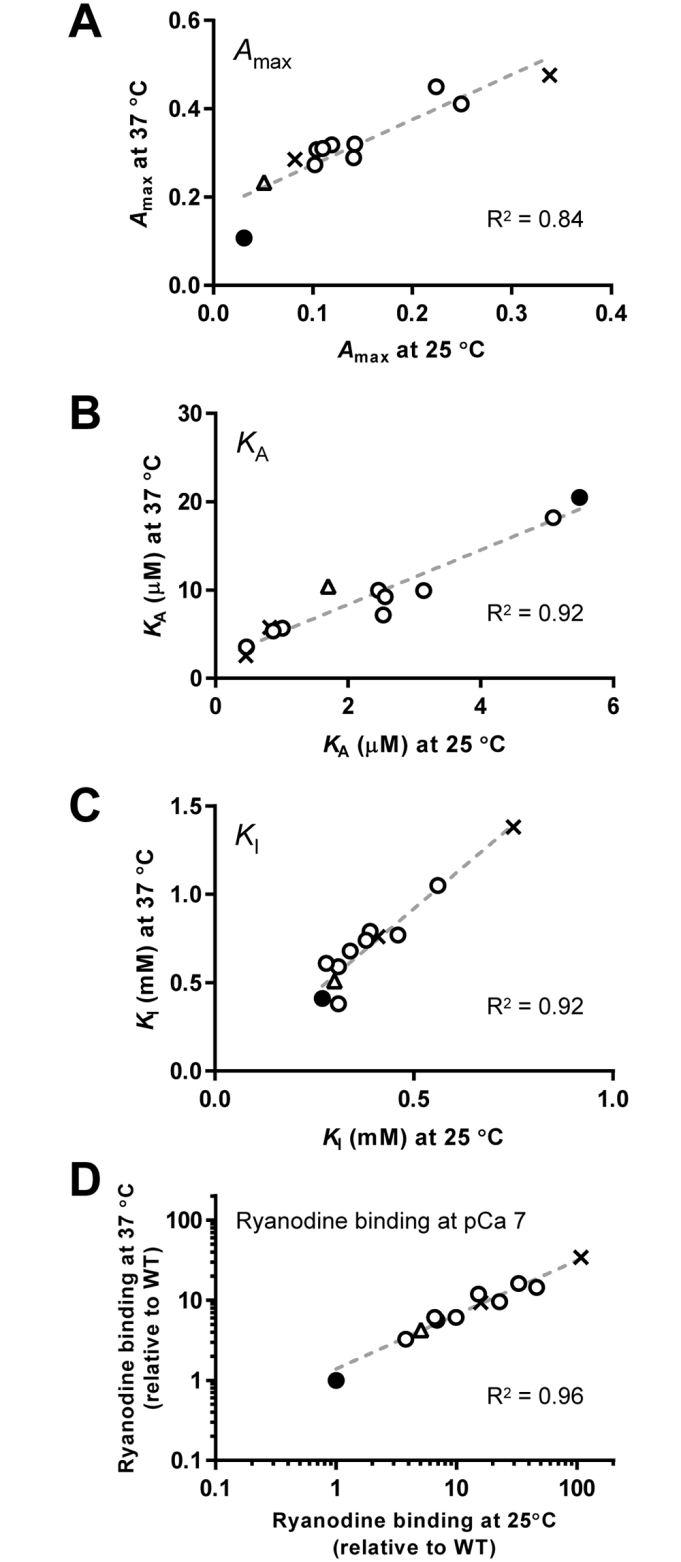
Comparison of the activity profiles between 25 and 37°C. The three parameters, *A*_max_ (**A**), *K*_A_ (**B**) and *K*_I_, (**C**) for WT (filled circles), MH (open circles), MH/CCD (crosses) and C36R (triangles) at 25 and 37°C are plotted. Note that high linear correlations (*R* ^2^ > 0.8) were found between mutants for all parameters. **D.** Estimated ryanodine binding at pCa 7.

We initially determined the Ca^2+^-dependent [^3^H]ryanodine binding of WT and mutant channels. Since the rate of [^3^H]ryanodine binding at 37°C was faster than at 25°C [39, 40], the incubation period was shortened to 3 h. Therefore, *B*/*B*_max_ values obtained at 37°C cannot be directly compared with those at 25°C. All the mutants showed greater binding than the WT and some mutants exhibited higher sensitivity to Ca^2+^ for activation at 37°C (Fig 6 and S1 Table). The effect of temperature on the activity profiles was examined by comparing the three parameters (*A*_max_, *K*_A_, and *K*_I_) for each mutant at 25 and 37°C (Fig 7A–7C). High linear correlations (*R* ^2^ > 0.8) were obtained between the mutants for the three parameters and no specific effects on C36R were detected. A good correlation was also observed for the estimated ryanodine binding at pCa 7 with the obtained parameters (Fig 7D). These findings indicate that temperature dependence of the activity profiles are similar between the N-terminal mutations examined.

**Fig 8:**
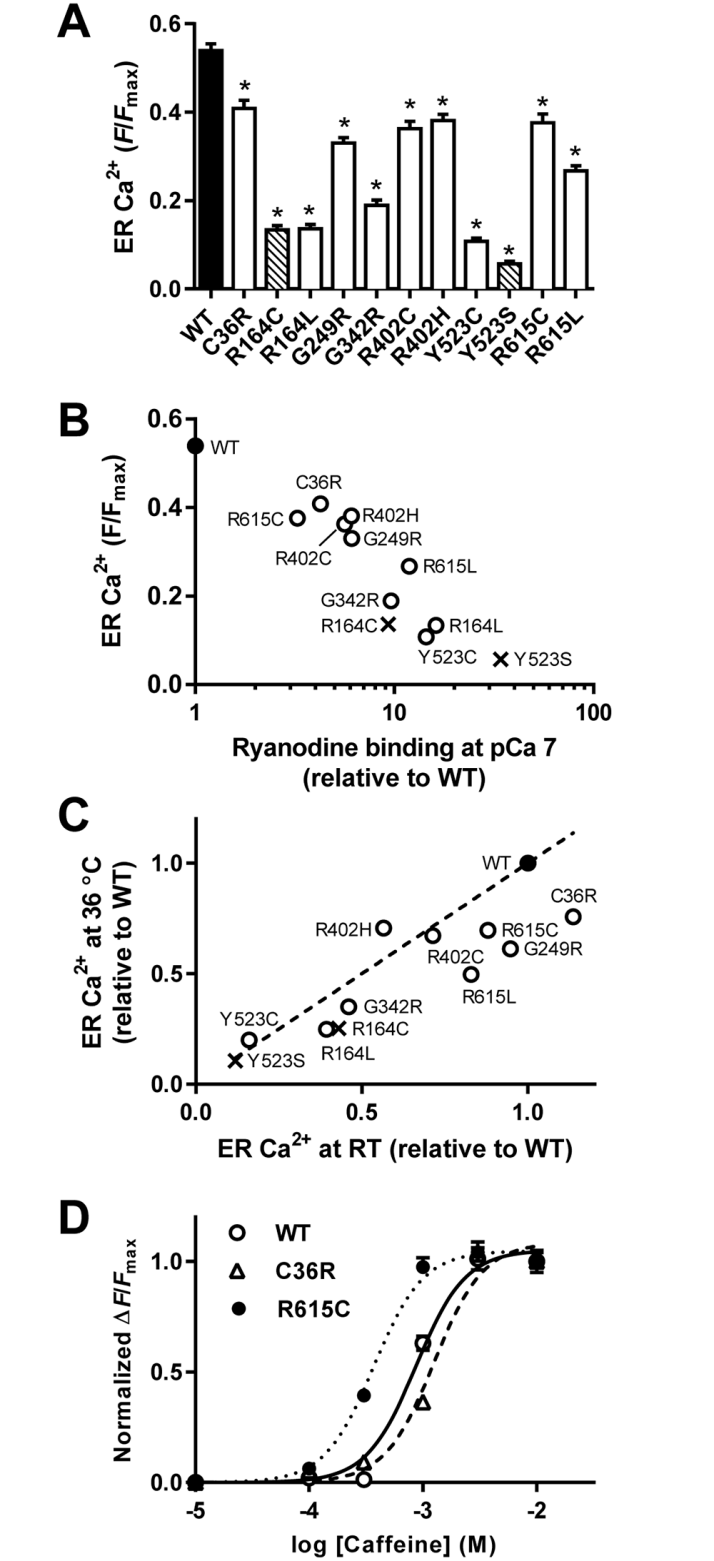
Cellular Ca^2+^ homeostasis of cells expressing WT or mutant RyR1s at 36°C. **A.** [Ca^2+^]_ER_ of WT (filled column), MH mutations (open columns) and MH/CCD mutations (hatched columns). Data are means ± SE (*n* = 30–67). *p < 0.05 compared with WT. **B.** [Ca^2+^]_ER_ for WT (filled circle), MH (open circles) and MH/CCD (crosses) were plotted against their estimated ryanodine binding at pCa 7 at 37°C. **C.** ER Ca^2+^ for MH (open circles) and MH/CCD (crosses) relative to WT (filled circle) at RT and 36°C was plotted. The dotted line represents y = x equation. Therefore, mutants under the line indicate that their ER Ca^2+^ relative to WT is lower at 36°C than at RT. **D.** Caffeine-induced Ca^2+^ transients of WT (open circles), C36R (open triangles) and R615C (filled circles) at 36°C. Note that caffeine sensitivity for C36R was not increased, whereas R615C exhibited a marked enhancement in caffeine sensitivity.

We next measured ER Ca^2+^ of HEK293 cells expressing mutant channels at 36°C. The ER Ca^2+^ level was reduced by varying amounts for the mutants when compared with the results of the WT (Fig 8A). C36R, which exhibited no significant change in ER Ca^2+^ at RT (see Fig 2), showed a significant decrease of ER Ca^2+^ at 36°C. The ER Ca^2+^ correlated well with the estimated ryanodine binding at pCa 7 at 37°C (Fig 8B). Linear correlations were obtained between ER Ca^2+^ at 36°C and RT. (Fig 8C), indicating that the severity order in terms of store depletion was similar between data measured at RT and 37°C. However, it is noteworthy that ER Ca^2+^ levels relative to WT was lower at 36°C than at RT for most mutants.

Since C36R exhibited no significant changes in caffeine sensitivity at RT (see Fig 1), we tested caffeine dependence of this mutant at 36°C. No increase in the caffeine sensitivity was observed (Fig 8D). In contrast, R615C, which showed a similar ER Ca^2+^ to C36R (Fig 8A), exhibited a marked increase in caffeine sensitivity at 36°C.

## Discussion

Characteristics of disease-associated mutations in the RyR1 channel have been evaluated using a number of methods. Assays for clinical practice, incorporating enhanced sensitivity to caffeine or halothane, for example the caffeine/halothane contracture test, have been used to detect MH using fresh biopsied muscles [13, 41–43]. However, comparison among various mutants is difficult with this method because data can be confounded by many factors, including gender and size of muscle preparations [13]. Heterologous expression systems with non-muscle [20, 30, 31] or muscle cells [14, 15, 44] allow the exploration of RyR1 mutants under the same conditions. These studies revealed different Ca^2+^ homeostasis between MH and MH/CCD mutations. However, the question of how the disease-associated mutations affect activity profiles of the RyR1 channel remains unanswered.

In this study, we generated HEK293 cells stably expressing 11 disease-associated mutations at 7 different positions in the N-terminal region. Pathological phenotype of the mutations were examined by cellular Ca^2+^ homeostasis and the activity profiles were determined using [^3^H]ryanodine binding and parameter analysis. The activity profiles are constituted by three parameters: Ca^2+^ sensitivities for activation (*K*_A_) and for inactivation (*K*_I_) and the gain (*A*_max_), an attainable maximum activity. We found that (1) the disease associated mutations affected *A*_max_ and *K*_A_ to varying extents in a site-specific manner, (2) channel activity at resting [Ca^2+^]_i_ estimated from the obtained parameters was highly variable among mutations, (3) the channel activity at resting [Ca^2+^]_i_ correlated well with [Ca^2+^]_ER_, and (4) temperature dependences of cellular Ca^2+^ homeostasis and activity profiles were similar between mutations. These results suggest that the disease-associated mutations in the N-terminal region divergently affect the activity profiles of the RyR1 channel to cause different disease phenotypes.

### Measurement of cellular Ca^2+^ homeostasis

Sensitivity to caffeine in caffeine-induced Ca^2+^ transients is a useful measure to detect MH. Caffeine makes RyR1 sensitive to activating Ca^2+^ [1, 2]. This triggers regenerative Ca^2+^ release, where Ca^2+^ released from the ER activates the channel [6, 7]. Most disease-associated mutations exhibited an enhanced sensitivity to caffeine (Fig 1). However, Y523C and Y523S showed unchanged and decreased sensitivities, respectively. Because the two mutants exhibited severe depletion of [Ca^2+^]_ER_ (Fig 2), the regenerative process would be weakened, leading to an apparent reduction in caffeine sensitivity. This may also explain why mutants with different channel activities showed similar EC_50_ values. Quantitative evaluation of caffeine sensitivity should be interpreted with caution in regard to [Ca^2+^]_ER_.

In the analysis of cellular Ca^2+^ homeostasis, [Ca^2+^]_ER_ is a good index for Ca^2+^ leakage, which is related to channel activity. [Ca^2+^]_ER_ for the disease-associated mutations has been estimated indirectly by measuring [Ca^2+^]_i_ upon stimulation with an agonist (e.g., carbachol or ATP) or after treatment with Ca^2+^-pump inhibitors (e.g., cyclopiazonic acid or thapsigargin) [14, 15, 31]. However, quantitative measurements were difficult in these studies because of the nonlinear relationship between [Ca^2+^]_ER_ and [Ca^2+^]_i_. Therefore, we directly measured [Ca^2+^]_ER_ using a novel genetically-encoded fluorescent Ca^2+^ indicator, R-CEPIA1er [24] (Fig 2). Quantitative measurements revealed a good correlation between [Ca^2+^]_ER_ and [^3^H]ryanodine binding at resting Ca^2+^ (Fig 5). [Ca^2+^]_ER_ is, thus, a useful measure for evaluating the activity of the RyR1 channel with disease-associated mutations.

We found that resting [Ca^2+^]_i_ is increased in cells expressing mutant channels (Fig 1). This is consistent with previous studies with heterologous expression systems [14, 30, 31, 45, 46] and knock-in mice [27, 39, 40, 47]. The [Ca^2+^]_i_ level is determined by a balance between influx and efflux of Ca^2+^ across the plasma membrane [48]. Recent studies demonstrated that store-operated Ca^2+^ entry (SOCE) pathway is accelerated in muscle cells from knock-in mice carrying R163C [49] and Y524S [50] mutations. SOCE pathway may also be involved in increase in resting [Ca^2+^]_i_ in HEK cell expressing RyR1 mutants.

### Properties of individual MH and MH/CCD mutations

Functional characterizations have been done for most mutations using a heterologous expression system (References in Table 1) or knock-in mice [27, 39, 40, 47, 51, 52]. The results of the present study combined with the previous reports provide information about properties of individual MH and MH/CCD mutations and explanation for different phenotypes between the mutations.

C36R exhibited small but significant [Ca^2+^]_ER_ depletion at 36°C, whereas it did not show any [Ca^2+^]_ER_ depletion at RT. The mutant channel exhibited small increase in the gain (*A*_max_) and the sensitivity to activating Ca^2+^ (1/*K*_A_), indicating a weak phenotype. Weak phenotype was suggested from the results with HEK293 cells [20, 31] and the presence of homozygous patients with the corresponding mutation [53]. We could not detect an enhanced caffeine sensitivity at RT nor 36°C, which was in contrast to the previous studies [20, 53]. It remains so far unclear about the reason for the difference.

R164C exhibited enhanced caffeine sensitivity with an increase in [Ca^2+^]_i_ and a depletion in [Ca^2+^]_ER_. This is consistent with previous studies with heterologous expression systems [14, 20, 31, 44–46] and knock-in mice carrying the corresponding mutation [27, 40]. R164L showed similar phenotypes. These mutations greatly enhanced the sensitivity to activating Ca^2+^ with a moderate increase in the gain, causing a large enhancement in channel activity.

G249R and G342R are located in the same domain [26]. The cellular Ca^2+^ homeostasis was more severely affected by G342R than by G249R. This difference was not clear in previous reports that compared the two mutations [20, 31]. The two mutations showed similar gain, but G342R exhibited a more enhanced sensitivity to activating Ca^2+^ than G249R. This may cause a more severe phenotype for G342R than for G249R.

R402C and R402H showed enhanced caffeine sensitivity with a moderate increase in [Ca^2+^]_i_ and a decrease in [Ca^2+^]_ER_. This is the first report to examine caffeine sensitivity and cellular [Ca^2+^] measurements for these mutations, although sensitivity to 4-chloro-m-cresol has been performed for R402C with HEK293 cells [54]. These mutations increased the gain with a moderate increase in the sensitivity to activating Ca^2+^.

Y523S exhibited the most severe phenotype in this study, which caused marked depletion in [Ca^2+^]_ER_ and an increase in [Ca^2+^]_i_. These properties are consistent with the previous findings with heterologous expression systems [14, 30, 31] and with knock-in mice carrying the corresponding mutation [39, 47, 51, 52]. Y523C, which has not been characterized so far, showed a similar phenotype. These mutations markedly increased both the gain and the sensitivity to activating Ca^2+^. We found that Y523S exhibited a more severe phenotype than R164C (Figs 1 and 2). This was also shown by the previous studies [14, 31]. The difference may be explained by the greater increase in the gain in Y523S. It would be interesting to address whether Y524S knock-in mice exhibits more severe phenotype than R163C knock-in mice.

R615C showed enhanced caffeine sensitivity, but no or only slight changes in [Ca^2+^]_i_ and [Ca^2+^]_ER_. This corresponds to previous reports with heterologous expression systems [15, 20, 30, 31]. This mutation greatly accelerated the gain with only slight effects on the Ca^2+^ sensitivity, consistent with the results with MH pigs carrying the corresponding mutation [55]. R615L showed a more severe phenotype than R615C, which is consistent with the results with biopsied muscles [56] or HEK293 cells [20, 31]. The greater increased gain and sensitivity to activating Ca^2+^ for R615L may explain the difference.

Overall, the results of the present study are consistent with previous findings and the activity profiles reasonably explain the differences in phenotypes among mutations. An increase in the gain is a common feature for all the mutations. The gain is important in the pathogenesis of the MH phenotype. In contrast, MH/CCD mutations exhibited a marked increase in sensitivity to activating Ca^2+^. An increased sensitivity to activating Ca^2+^ further accelerates channel activity to exceed the threshold for CICR to trigger Ca^2+^ leakage from Ca^2+^ stores under resting conditions.

### Effects of temperature on cellular Ca^2+^ homeostasis and activity profiles

It has been hypothesized that some specific mutations, especially C36R, may destabilize the channel in a temperature-dependent manner and at temperatures close to 37°C. This is based on the finding that melting points for the N-terminal domain of several mutant RyR1s was significantly reduced [38]. However, the temperature dependence on activity profiles was similar among the mutants and no specific effects were observed for C36R or other mutants (Fig 7). Our data may not support the above hypothesis at least for the mutations examined in this study.

The heterozygous knock-in mice for MH/CCD demonstrate heat-induced muscle contracture and sudden death [27, 28]. Heat-induced activation of the mutant RyR1 channel is proposed to cause Ca^2+^ release from the SR under resting conditions [39, 40, 47]. ER Ca^2+^ relative to WT was lower at 36°C than at RT for most mutants, indicating an increase in Ca^2+^ leakage from the ER (Fig 8). Heat-induced activation may be a common characteristic of the N-terminal RyR1 mutants examined.

### Physiological and pathological significance

The combination of [^3^H]ryanodine binding and parameter analysis provided a relative rank order of the activity of the mutant channels. The rank order may predict risk and severity of the diseases. This is highly helpful for diagnosis of the diseases using genetic analysis. Interestingly, three MH mutations (R164L, G342R and Y523C) exhibited a high channel activity and severe depletion of [Ca^2+^]_ER_, which is comparable to that seen for MH/CCD mutations (R164C and Y523S) (Fig 5). It is expected that these mutations may also show signs of MH/CCD. Further evaluation of the muscle phenotype in patients with these mutations would be particularly interesting.

The activity profiles also provide useful information about treatment of the diseases. Currently, there is no specific treatment for CCD. An enhanced sensitivity to activating Ca^2+^ greatly contributes to Ca^2+^ leakage from Ca^2+^ stores in MH/CCD mutations (Figs 4 and 5). Certain drugs that decrease the sensitivity to activating Ca^2+^ may inhibit Ca^2+^ leakage and thus represent a potential candidate for treatment of CCD.

A recent crystal structure of RyR1 has revealed that many mutations are located on the domain-domain interfaces at the N-terminal region [26, 38]. However it remains unclear how postulated alterations in inter-domain interaction cause acceleration of channel activity or increase in Ca^2+^ sensitivity. The activity profiles of the mutant channels would provide important information about structure-function relationships of the disease-associated mutations of the RyR1 channel.

It has been proposed that a reduced threshold level of [Ca^2+^]_ER_ for spontaneous Ca^2+^ release underlies a causal mechanism of MH carrying the R615C mutation [32]. We confirmed their finding that R615C reduced the threshold for caffeine-induced Ca^2+^ oscillations compared with WT (Fig 4). However, other mutants did not show caffeine-induced Ca^2+^ oscillations. Thus, occurrence of Ca^2+^ oscillations may not be a common characteristic for the MH and MH/CCD mutations. Further investigations will address possible contribution of luminal Ca^2+^ regulation to the underlying mechanism of MH and MH/CCD carrying the disease-associated mutations other than R615C.

### Limitations and future research

In skeletal muscle, the activity of RyR1 is controlled by DHPR and can be modulated by skeletal muscle-specific proteins [57, 58]. Since HEK293 cells lack such modulators, it can be argued that RyR1 expressed in HEK293 cells is somehow different from that in skeletal muscle cells. However, the three parameters, *A*_max_, *K*_A_ and *K*_I_, for WT RyR1 channels were very similar to those of skeletal muscle [36, 37]. In addition, the activity profiles of R164C and R615C are in good agreement with those from animals carrying the same mutations (R163C knock-in mice [40] and MH pigs carrying R615C [55]). Thus, the channel activities of RyR1 mutants expressed in HEK293 cells are likely to reflect that in skeletal muscle.

Most human MH and CCD patients are heterozygous for WT and mutant alleles [9]. This is in contrast to our recombinant HEK293 cells that have only the mutant gene. It is reasonable to expect that the phenotype in our study is more severe compared with that seen in patients. In addition, properties of the heterotetrameric channels composed of WT and mutant subunits are, as yet, unclear. However, phenotype severity determined by a cell culture system [14, 15, 20, 30, 31, 44] corresponds to that of biopsied muscle from patients [13, 41–43]. The rank order of channel activity in this study is also consistent with that from patients. It is therefore expected that the properties of homotetrameric mutant channels are qualitatively similar to those of heterotetrameric channels.

Among the reported MH and MH/CCD mutations, many have been discovered in one or small numbers of families [9]. In such a situation, the effect of genetic background may be high and this makes it difficult to compare severity between mutations. The present approach, consisting of cellular Ca^2+^ homeostasis measurements and determination of activity profiles, provides useful information about phenotype and disease severity caused by individual mutations. Construction of a database for this information should aid diagnosis and predict prognosis of the diseases.

## Supporting Information

S1 Fig Simulation of Ca^2+^-dependent channel activity and activity profiles. **A.** Ca^2+^-dependent channel activity (bold line) and fractions of A-site occupied by Ca^2+^ (*f*_A_, thin line) and of I-site free from Ca^2+^ (1–*f*_I_, dashed line) were simulated by eqs (1)–(3) using the following parameters: *A*_max_ = 0.05, *K*_A_ = 10 μM, *n*_A_ = 1.2, *K*_I_ = 0.15 mM, and *n*_I_ = 1.5. **B.** Effect of individual parameters on channel activity. Parameters for the original curve (black line) are the same as those in **A.** Channel activity was simulated with either 4-fold decreased *K*_A_ (*K*_A_ = 2.5 μM, left), 4-fold increased *A*_max_ (*A*_max_ = 0.2, center), or 4-fold increased *K*_I_ (*K*_I_ = 0.6 mM, right). **C.** The activity profiles of WT and mutant channels in **B.** The three parameters, *A*_max_, 1/*K*_A_ and *K*_I_, were plotted on the radar charts relative to WT. 1/*K*_A_ was used as the parameter for activating Ca^2+^ dissociation constants, in which a larger value represents higher sensitivity.
(PDF)

S2 Fig Expression of mutant RyR1s in HEK293 cells. Western blot analysis of RyR1 in microsomes from HEK293 cells expressing WT or disease-associated mutants. The mutant RyR1s showed gel mobility similar to that of the WT. Calnexin was used as a loading control.
(PDF)

S1 Table Parameters for Ca^2+^-dependent [^3^H]ryanodine binding. Data are mean ± SE (*n* = 3–5). Numbers in parentheses indicate fold change of the parameters relative to WT. *P < 0.05 vs. WT.
(DOC)

## References

[1] Meissner G. Ryanodine receptor/Ca^2+^ release channels and their regulation by endogenous effectors. Annu Rev Physiol. 1994;56:485–508. 7516645 10.1146/annurev.ph.56.030194.002413

[2] Ogawa Y. Role of ryanodine receptors. Crit Rev Biochem Mol Biol. 1994;29:229–74. 8001396 10.3109/10409239409083482

[3] Franzini-Armstrong C, Protasi F. Ryanodine receptors of striated muscles: a complex channel capable of multiple interactions. Physiol Rev. 1997;77(3):699–729. 9234963 10.1152/physrev.1997.77.3.699

[4] Rios E, Pizarro G. Voltage sensor of excitation-contraction coupling in skeletal muscle. Physiol Rev. 1991;71(3):849–908. 2057528 10.1152/physrev.1991.71.3.849

[5] Schneider MF. Control of calcium release in functioning skeletal muscle fibers. Annu Rev Physiol. 1994;56:463–84. 8010748 10.1146/annurev.ph.56.030194.002335

[6] Endo M. Calcium release from the sarcoplasmic reticulum. Physiol Rev. 1977;57:71–108. 13441 10.1152/physrev.1977.57.1.71

[7] Endo M. Calcium-induced calcium release in skeletal muscle. Physiol Rev. 2009;89(4):1153–76. 10.1152/physrev.00040.2008 19789379

[8] Murayama T, Kurebayashi N. Two ryanodine receptor isoforms in nonmammalian vertebrate skeletal muscle: possible roles in excitation-contraction coupling and other processes. Prog Biophys Mol Biol. 2011;105(3):134–44. Epub 2010/10/30. 10.1016/j.pbiomolbio.2010.10.003 . 21029746

[9] Robinson R, Carpenter D, Shaw MA, Halsall J, Hopkins P. Mutations in RYR1 in malignant hyperthermia and central core disease. Hum Mutat. 2006;27(10):977–89. . 16917943 10.1002/humu.20356

[10] Hopkins PM. Malignant hyperthermia: advances in clinical management and diagnosis. Br J Anaesth. 2000;85(1):118–28. Epub 2000/08/06. . 10928000

[11] Lanner JT, Georgiou DK, Joshi AD, Hamilton SL. Ryanodine receptors: structure, expression, molecular details, and function in calcium release. Cold Spring Harb Perspect Biol. 2010;2(11):a003996 Epub 2010/10/22. 10.1101/cshperspect.a003996 20961976 PMC2964179

[12] Treves S, Jungbluth H, Muntoni F, Zorzato F. Congenital muscle disorders with cores: the ryanodine receptor calcium channel paradigm. Curr Opin Pharmacol. 2008;8(3):319–26. Epub 2008/03/04. 10.1016/j.coph.2008.01.005 . 18313359

[13] Robinson RL, Brooks C, Brown SL, Ellis FR, Halsall PJ, Quinnell RJ, et al RYR1 mutations causing central core disease are associated with more severe malignant hyperthermia in vitro contracture test phenotypes. Hum Mutat. 2002;20(2):88–97. Epub 2002/07/19. 10.1002/humu.10098 . 12124989

[14] Avila G, Dirksen RT. Functional effects of central core disease mutations in the cytoplasmic region of the skeletal muscle ryanodine receptor. J Gen Physiol. 2001;118(3):277–90. Epub 2001/08/29. 11524458 10.1085/jgp.118.3.277 PMC2229502

[15] Dirksen RT, Avila G. Distinct effects on Ca^2+^ handling caused by malignant hyperthermia and central core disease mutations in RyR1. Biophys J. 2004;87(5):3193–204. . 15347586 10.1529/biophysj.104.048447 PMC1304789

[16] Avila G, O'Brien JJ, Dirksen RT. Excitation--contraction uncoupling by a human central core disease mutation in the ryanodine receptor. Proceedings of the National Academy of Sciences of the United States of America. 2001;98(7):4215–20. Epub 2001/03/29. 10.1073/pnas.071048198 11274444 PMC31205

[17] Dirksen RT, Avila G. Altered ryanodine receptor function in central core disease: leaky or uncoupled Ca^2+^ release channels? Trends Cardiovasc Med. 2002;12(5):189–97. . 12161072 10.1016/s1050-1738(02)00163-9

[18] Avila G, O'Connell KM, Dirksen RT. The pore region of the skeletal muscle ryanodine receptor is a primary locus for excitation-contraction uncoupling in central core disease. The Journal of general physiology. 2003;121(4):277–86. Epub 2003/03/19. 10.1085/jgp.200308791 12642598 PMC2217374

[19] Murayama T, Ogawa Y. Roles of two ryanodine receptor isoforms coexisting in skeletal muscle. Trends Cardiovasc Med. 2002;12(7):305–11. . 12458093 10.1016/s1050-1738(02)00179-2

[20] Tong J, Oyamada H, Demaurex N, Grinstein S, McCarthy TV, MacLennan DH. Caffeine and halothane sensitivity of intracellular Ca^2+^ release is altered by 15 calcium release channel (ryanodine receptor) mutations associated with malignant hyperthermia and/or central core disease. J Biol Chem. 1997;272(42):26332–9. 9334205 10.1074/jbc.272.42.26332

[21] Murayama T, Kurebayashi N, Oba T, Oyamada H, Oguchi K, Sakurai T, et al Role of amino-terminal half of the S4-S5 linker in type 1 ryanodine receptor (RyR1) channel gating. J Biol Chem. 2011;286(41):35571–7. Epub 2011/08/25. 10.1074/jbc.M111.255240 21862589 PMC3195593

[22] Grynkiewicz G, Poenie M, Tsien RY. A new generation of Ca^2+^ indicators with greatly improved fluorescence properties. J Biol Chem. 1985;260(6):3440–50. Epub 1985/03/25. . 3838314

[23] Zhao Y, Araki S, Wu J, Teramoto T, Chang YF, Nakano M, et al An expanded palette of genetically encoded Ca^2+^ indicators. Science. 2011;333(6051):1888–91. Epub 2011/09/10. 10.1126/science.1208592 21903779 PMC3560286

[24] Suzuki J, Kanemaru K, Ishii K, Ohkura M, Okubo Y, Iino M. Imaging intraorganellar Ca^2+^ at subcellular resolution using CEPIA. Nat Commun. 2014;5:4153 10.1038/ncomms5153 . 24923787 PMC4082642

[25] Bers DM, Patton CW, Nuccitelli R. A practical guide to the preparation of Ca(^2+^) buffers. Methods in cell biology. 2010;99:1–26. 10.1016/B978-0-12-374841-6.00001-3 . 21035681

[26] Tung CC, Lobo PA, Kimlicka L, Van Petegem F. The amino-terminal disease hotspot of ryanodine receptors forms a cytoplasmic vestibule. Nature. 2010;468(7323):585–8. Epub 2010/11/05. 10.1038/nature09471 . 21048710

[27] Yang T, Riehl J, Esteve E, Matthaei KI, Goth S, Allen PD, et al Pharmacologic and functional characterization of malignant hyperthermia in the R163C RyR1 knock-in mouse. Anesthesiology. 2006;105(6):1164–75. . 17122579 10.1097/00000542-200612000-00016

[28] Chelu MG, Goonasekera SA, Durham WJ, Tang W, Lueck JD, Riehl J, et al Heat- and anesthesia-induced malignant hyperthermia in an RyR1 knock-in mouse. FASEB J. 2006;20(2):329–30. 10.1096/fj.05-4497fje . 16284304

[29] Fujii J, Otsu K, Zorzato F, de Leon S, Khanna VK, Weiler JE, et al Identification of a mutation in porcine ryanodine receptor associated with malignant hyperthermia. Science. 1991;253(5018):448–51. . 1862346 10.1126/science.1862346

[30] Brini M, Manni S, Pierobon N, Du GG, Sharma P, MacLennan DH, et al Ca^2+^ signaling in HEK-293 and skeletal muscle cells expressing recombinant ryanodine receptors harboring malignant hyperthermia and central core disease mutations. J Biol Chem. 2005;280(15):15380–9. . 15689621 10.1074/jbc.M410421200

[31] Tong J, McCarthy TV, MacLennan DH. Measurement of resting cytosolic Ca^2+^ concentrations and Ca^2+^ store size in HEK-293 cells transfected with malignant hyperthermia or central core disease mutant Ca^2+^ release channels. J Biol Chem. 1999;274(2):693–702. 9873004 10.1074/jbc.274.2.693

[32] Jiang D, Chen W, Xiao J, Wang R, Kong H, Jones PP, et al Reduced threshold for luminal Ca^2+^ activation of RyR1 underlies a causal mechanism of porcine malignant hyperthermia. J Biol Chem. 2008;283(30):20813–20. 10.1074/jbc.M801944200 18505726 PMC2475718

[33] Chu A, Diaz-Munos M, Hawkes MJ, Brush K, Hamilton S. Ryanodine as a probe for the functional state of the skeletal muscle sarcoplasmic reticulum calcium release channel. Mol Pharmacol. 1990;37:735–41. 1692609

[34] Meissner G, El-Hashem A. Ryanodine as a functional probe of the skeletal muscle sarcoplasmic reticulum Ca^2+^ release channel. Mol Cell Biochem. 1992;114:119–23. 1334225 10.1007/BF00240306

[35] Murayama T, Kurebayashi N, Ogawa Y. Role of Mg^2+^ in Ca^2+^-induced Ca^2+^ release through ryanodine receptors of frog skeletal muscle: Modulations by adenine nucleotides and caffeine. Biophys J. 2000;78:1810–24. 10733962 10.1016/S0006-3495(00)76731-2 PMC1300776

[36] Murayama T, Oba T, Kobayashi S, Ikemoto N, Ogawa Y. Postulated role of interdomain interactions within the type 1 ryanodine receptor in the low gain of Ca^2+^-induced Ca^2+^ release activity of mammalian skeletal muscle sarcoplasmic reticulum. Am J Physiol Cell Physiol. 2005;288(6):C1222–C30. 15677376 10.1152/ajpcell.00415.2004

[37] Murayama T, Ogawa Y. RyR1 exhibits lower gain of CICR activity than RyR3 in the SR: evidence for selective stabilization of RyR1 channel. Am J Physiol Cell Physiol. 2004;287(1):C36–C45. 14985235 10.1152/ajpcell.00395.2003

[38] Kimlicka L, Lau K, Tung CC, Van Petegem F. Disease mutations in the ryanodine receptor N-terminal region couple to a mobile intersubunit interface. Nat Commun. 2013;4:1506 Epub 2013/02/21. 10.1038/ncomms2501 23422674 PMC3586727

[39] Durham WJ, Aracena-Parks P, Long C, Rossi AE, Goonasekera SA, Boncompagni S, et al RyR1 S-nitrosylation underlies environmental heat stroke and sudden death in Y522S RyR1 knockin mice. Cell. 2008;133(1):53–65. Epub 2008/04/09. 10.1016/j.cell.2008.02.042 18394989 PMC2366094

[40] Feng W, Barrientos GC, Cherednichenko G, Yang T, Padilla IT, Truong K, et al Functional and biochemical properties of ryanodine receptor type 1 channels from heterozygous R163C malignant hyperthermia-susceptible mice. Mol Pharmacol. 2011;79(3):420–31. Epub 2010/12/16. 10.1124/mol.110.067959 21156754 PMC3061367

[41] Fiege M, Wappler F, Weisshorn R, Ulrich Gerbershagen M, Steinfath M, Schulte Am Esch J. Results of contracture tests with halothane, caffeine, and ryanodine depend on different malignant hyperthermia-associated ryanodine receptor gene mutations. Anesthesiology. 2002;97(2):345–50. Epub 2002/08/02. . 12151923 10.1097/00000542-200208000-00010

[42] Monnier N, Kozak-Ribbens G, Krivosic-Horber R, Nivoche Y, Qi D, Kraev N, et al Correlations between genotype and pharmacological, histological, functional, and clinical phenotypes in malignant hyperthermia susceptibility. Hum Mutat. 2005;26(5):413–25. Epub 2005/09/16. 10.1002/humu.20231 . 16163667

[43] Carpenter D, Robinson RL, Quinnell RJ, Ringrose C, Hogg M, Casson F, et al Genetic variation in RYR1 and malignant hyperthermia phenotypes. Br J Anaesth. 2009;103(4):538–48. Epub 2009/08/04. 10.1093/bja/aep204 . 19648156

[44] Yang T, Ta TA, Pessah IN, Allen PD. Functional defects in six ryanodine receptor isoform-1 (RyR1) mutations associated with malignant hyperthermia and their impact on skeletal excitation-contraction coupling. J Biol Chem. 2003;278(28):25722–30. 12732639 10.1074/jbc.M302165200

[45] Yang T, Esteve E, Pessah IN, Molinski TF, Allen PD, Lopez JR. Elevated resting [Ca^2+^]i in myotubes expressing malignant hyperthermia RyR1 cDNAs is partially restored by modulation of passive calcium leak from the SR. Am J Physiol Cell Physiol. 2007;292(5):C1591–8. Epub 2006/12/22. 10.1152/ajpcell.00133.2006 . 17182726

[46] Nakano M, Oyamada H, Yamazawa T, Murayama T, Nanba H, Iijima K, et al Construction and expression of ryanodine receptor mutants relevant to malignant hyperthermia patients in Japan. Showa Univ J Med Sci. 2014;26(1):27–38.

[47] Lanner JT, Georgiou DK, Dagnino-Acosta A, Ainbinder A, Cheng Q, Joshi AD, et al AICAR prevents heat-induced sudden death in RyR1 mutant mice independent of AMPK activation. Nat Med. 2012;18(2):244–51. Epub 2012/01/11. 10.1038/nm.2598 22231556 PMC3274651

[48] Rios E. The cell boundary theorem: a simple law of the control of cytosolic calcium concentration. The journal of physiological sciences: JPS. 2010;60(1):81–4. 10.1007/s12576-009-0069-z 19937486 PMC2821834

[49] Eltit JM, Ding X, Pessah IN, Allen PD, Lopez JR. Nonspecific sarcolemmal cation channels are critical for the pathogenesis of malignant hyperthermia. FASEB J. 2013;27(3):991–1000. 10.1096/fj.12-218354 23159934 PMC3574284

[50] Yarotskyy V, Protasi F, Dirksen RT. Accelerated activation of SOCE current in myotubes from two mouse models of anesthetic- and heat-induced sudden death. PLoS One. 2013;8(10):e77633 10.1371/journal.pone.0077633 24143248 PMC3797063

[51] Chelu MG, Danila CI, Gilman CP, Hamilton SL. Regulation of ryanodine receptors by FK506 binding proteins. Trends Cardiovasc Med. 2004;14(6):227–34. . 15451514 10.1016/j.tcm.2004.06.003

[52] Manno C, Figueroa L, Royer L, Pouvreau S, Lee CS, Volpe P, et al Altered Ca^2+^ concentration, permeability and buffering in the myofibre Ca^2+^ store of a mouse model of malignant hyperthermia. J Physiol. 2013;591(Pt 18):4439–57. 10.1113/jphysiol.2013.259572 23798496 PMC3784192

[53] Lynch PJ, Krivosic-Horber R, Reyford H, Monnier N, Quane K, Adnet P, et al Identification of heterozygous and homozygous individuals with the novel RYR1 mutation Cys35Arg in a large kindred. Anesthesiology. 1997;86(3):620–6. Epub 1997/03/01. . 9066328 10.1097/00000542-199703000-00014

[54] Sato K, Roesl C, Pollock N, Stowell KM. Skeletal muscle ryanodine receptor mutations associated with malignant hyperthermia showed enhanced intensity and sensitivity to triggering drugs when expressed in human embryonic kidney cells. Anesthesiology. 2013;119(1):111–8. 10.1097/ALN.0b013e31828cebfe . 23459219

[55] Murayama T, Oba T, Hara H, Wakebe K, Ikemoto N, Ogawa Y. Postulated role of interdomain interaction between regions 1 and 2 within type 1 ryanodine receptor in the pathogenesis of porcine malignant hyperthermia. Biochem J. 2007;402(2):349–57. . 17107340 10.1042/BJ20061040 PMC1798429

[56] Quane KA, Ording H, Keating KE, Manning BM, Heine R, Bendixen D, et al Detection of a novel mutation at amino acid position 614 in the ryanodine receptor in malignant hyperthermia. Br J Anaesth. 1997;79(3):332–7. Epub 1997/12/09. . 9389851 10.1093/bja/79.3.332

[57] Franzini-Armstrong C, Jorgensen AO. Structure and development of E-C coupling units in skeletal muscle. Annu Rev Physiol. 1994;56:509–34. 8010750 10.1146/annurev.ph.56.030194.002453

[58] Sutko JL, Airey JA. Ryanodine receptor Ca^2+^ release channels: does diversity in form equal diversity in function? Physiol Rev. 1996;76:1027–71. 8874493 10.1152/physrev.1996.76.4.1027

[59] Sato K, Pollock N, Stowell KM. Functional studies of RYR1 mutations in the skeletal muscle ryanodine receptor using human RYR1 complementary DNA. Anesthesiology. 2010;112(6):1350–4. 10.1097/ALN.0b013e3181d69283 . 20461000

