# Supplementary material for: Divergent Activity Profiles of Type 1 Ryanodine Receptor Channels Carrying Malignant Hyperthermia and Central Core Disease Mutations in the Amino-Terminal Region
Source: PLoS One. 2015 Jun 26;10(6):e0130606. doi: 10.1371/journal.pone.0130606 (PMC4482644; doi:10.1371/journal.pone.0130606)

S1 Fig. Simulation of Ca^{2+}-dependent channel activity and activity profiles. A. Ca^{2+}-dependent channel activity (bold line) and fractions of A-site occupied by Ca^{2+} (f_A, thin line) and of I-site free from Ca^{2+} ($1\text{-}f_I$, dashed line) were simulated by equations (1)–(3) using the following parameters: $A_{max} = 0.05$, $K_A = 10$ μM, and $K_I = 0.15$ mM. n_A and n_I were fixed at 1.0. **B.** Effect of individual parameters on channel activity. Parameters for the original curve (black line) are the same as those in **A**. Channel activity was simulated with either 4-fold decreased K_A ($K_A = 2.5$ μM, left), 4-fold increased A_{max} ($A_{max} = 0.2$, center), or 4-fold increased K_I ($K_I = 0.6$ mM, right). **C.** Activity profiles of WT and mutant channels in **B**. The three parameters, A_{max}, $1/K_A$ and K_I, were plotted on the radar charts relative to WT. $1/K_A$ was used as the parameter for activating Ca^{2+} dissociation constants, in which a larger value represents higher sensitivity.

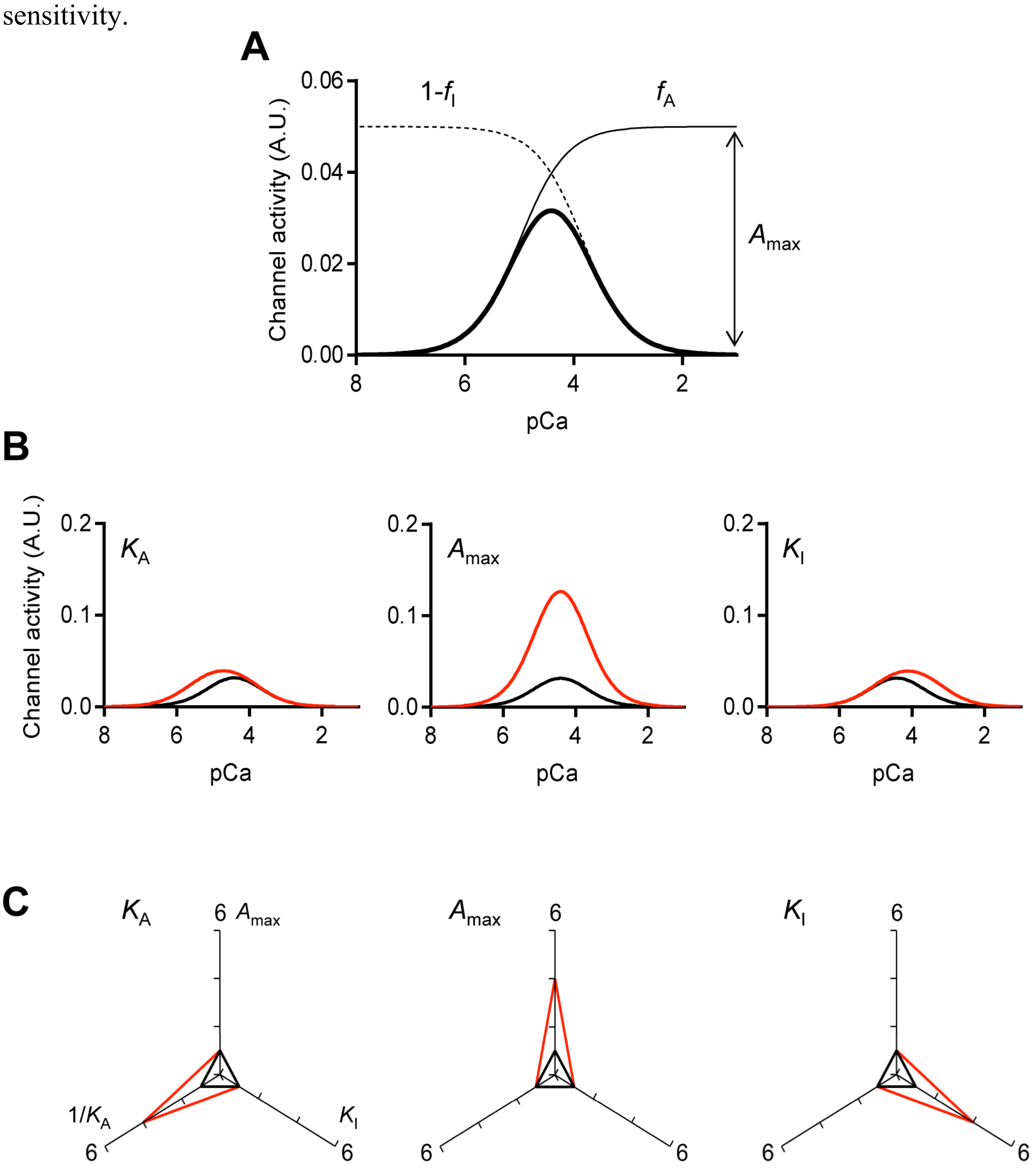

Supplement: S1 Fig — A. Ca2+-dependent channel activity (bold line) and fractions of A-site occupied by Ca2+ (f A, thin line) and of I-site free from Ca2+ (1–f I, dashed line) were simulated by eqs (1)–(3) using the following parameters: A max = 0.05, K A = 10 μM, n A = 1.2, K I = 0.15 mM, and n I = 1.5. B. Effect of individual parameters on channel activity. Parameters for the original curve (black line) are the same as those in A. Channel activity was simulated with either 4-fold decreased K A (K A = 2.5 μM, left), 4-fold increased A max (A max = 0.2, center), or 4-fold increased K I (K I = 0.6 mM, right). C. The activity profiles of WT and mutant channels in B. The three parameters, A max, 1/K A and K I, were plotted on the radar charts relative to WT. 1/K A was used as the parameter for activating Ca2+ dissociation constants, in which a larger value represents higher sensitivity. (PDF) [file pone.0130606.s001.pdf]
