# Supplementary material for: Divergent Activity Profiles of Type 1 Ryanodine Receptor Channels Carrying Malignant Hyperthermia and Central Core Disease Mutations in the Amino-Terminal Region
Source: PLoS One. 2015 Jun 26;10(6):e0130606. doi: 10.1371/journal.pone.0130606 (PMC4482644; doi:10.1371/journal.pone.0130606)

S2 Fig. Expression of mutant RyR1s in HEK293 cells. Western blot analysis of RyR1 in microsomes from HEK293 cells expressing WT or disease-associated mutants. The mutant RyR1s showed gel mobility similar to that of the WT. Calnexin was used as a loading control.

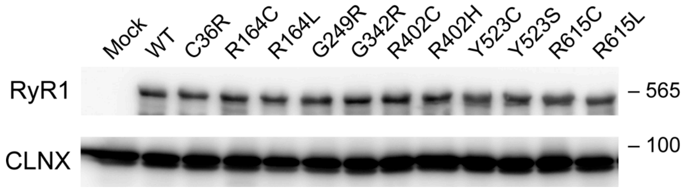

Supplement: S2 Fig — Western blot analysis of RyR1 in microsomes from HEK293 cells expressing WT or disease-associated mutants. The mutant RyR1s showed gel mobility similar to that of the WT. Calnexin was used as a loading control. (PDF) [file pone.0130606.s002.pdf]
