# Supplementary material for: Divergent Activity Profiles of Type 1 Ryanodine Receptor Channels Carrying Malignant Hyperthermia and Central Core Disease Mutations in the Amino-Terminal Region
Source: PLoS One. 2015 Jun 26;10(6):e0130606. doi: 10.1371/journal.pone.0130606 (PMC4482644; doi:10.1371/journal.pone.0130606)
Supplement: S1 Table — Data are mean ± SE (n = 3–5). Numbers in parentheses indicate fold change of the parameters relative to WT. *P < 0.05 vs. WT. (DOC) [file pone.0130606.s003.doc]

**S1 Table. Parameters for Ca^2+^-dependent [^3^H]ryanodine binding.**

| **Mutation** | **T (**°C**)** | ***A*_max_** | ***K*_A_ (µM)** | ***K*_I_ (mM)** |
| --- | --- | --- | --- | --- |
| WT | 25  37 | 0.031 ± 0.002 (1.0)  0.107 ± 0.006 (1.0) | 5.5 ± 0.9 (1.0)  20.5 ± 2.5 (1.0) | 0.27 ± 0.03 (1.0)  0.41 ± 0.04 (1.0) |
| C36R | 25  37 | 0.051 ± 0.002^*^ (1.6)  0.233 ± 0.007^*^ (2.2) | 1.7 ± 0.2^*^ (0.31)  10.4 ± 0.9^*^ (0.51) | 0.30 ± 0.03 (1.1)  0.51 ± 0.04 (1.3) |
| R164C | 25  37 | 0.082 ± 0.003^*^ (2.6)  0.285 ± 0.005^*^ (2.7) | 0.82 ± 0.09^*^ (0.15)  5.8 ± 0.4^*^ (0.28) | 0.41 ± 0.04^*^ (1.5)  0.76 ± 0.04^*^ (1.9) |
| R164L | 25  37 | 0.104 ± 0.003^*^ (3.3)  0.307 ± 0.006^*^ (2.9) | 0.47 ± 0.05^*^ (0.09)  3.6 ± 0.3^*^ (0.17) | 0.39 ± 0.04 (1.4)  0.79 ± 0.05^*^ (2.0) |
| G249R | 25  37 | 0.142 ± 0.002^*^ (4.5)  0.320 ± 0.012^*^ (3.0) | 2.5 ± 0.1^*^ (0.45)  10.0 ± 1.1^*^ (0.49) | 0.34 ± 0.02 (1.3)  0.68 ± 0.06^*^ (1.7) |
| G342R | 25  37 | 0.141 ± 0.003^*^ (4.5)  0.289 ± 0.006^*^ (2.7) | 1.0 ± 0.1^*^ (0.18)  5.7 ± 0.4^*^ (0.28) | 0.38 ± 0.03 (1.4)  0.74 ± 0.04^*^ (1.8) |
| R402C | 25  37 | 0.102 ± 0.002^*^ (3.3)  0.273 ± 0.006^*^ (2.5) | 2.6 ± 0.1^*^ (0.47)  9.2 ± 0.6^*^ (0.45) | 0.31 ± 0.02 (1.1)  0.59 ± 0.03 (1.5) |
| R402H | 25  37 | 0.119 ± 0.003^*^ (3.8)  0.318 ± 0.008^*^ (3.0) | 3.1 ± 0.2^*^ (0.57)  9.9 ± 0.7^*^ (0.49) | 0.28 ± 0.02 (1.0)  0.61 ± 0.04 (1.5) |
| Y523C | 25  37 | 0.249 ± 0.005^*^ (8.0)  0.411 ± 0.009^*^ (3.8) | 0.87 ± 0.06^*^ (0.16)  5.4 ± 0.5^*^ (0.26) | 0.56 ± 0.03^*^ (2.1)  1.05 ± 0.07^*^ (2.6) |
| Y523S | 25  37 | 0.338 ± 0.005^*^ (10.8)  0.476 ± 0.012^*^ (4.4) | 0.46 ± 0.03^*^ (0.08)  2.6 ± 0.3^*^ (0.13) | 0.75 ± 0.04^*^ (2.8)  1.38 ± 0.13^*^ (3.4) |
| R615C | 25  37 | 0.110 ± 0.003^*^ (3.5)  0.310 ± 0.018^*^ (2.9) | 5.1 ± 0.5 (0.93)  18.2 ± 2.4 (0.89) | 0.31 ± 0.02 (1.2)  0.38 ± 0.04 (0.9) |
| R615L | 25  37 | 0.224 ± 0.003^*^ (7.1)  0.450 ± 0.011^*^ (4.2) | 2.5 ± 0.1^*^ (0.46)  7.2 ± 0.6^*^ (0.35) | 0.46 ± 0.02^*^ (1.7)  0.77 ± 0.05^*^ (1.9) |

Data are mean ± SE (*n* = 3–5).

Numbers in parentheses indicate fold change of the parameters relative to WT.

^*^*P* < 0.05 vs. WT
